# Calcitriol and Its Analogs Establish the Immunosuppressive Microenvironment That Drives Metastasis in 4T1 Mouse Mammary Gland Cancer

**DOI:** 10.3390/ijms19072116

**Published:** 2018-07-20

**Authors:** Agata Pawlik, Artur Anisiewicz, Beata Filip-Psurska, Marcin Nowak, Eliza Turlej, Justyna Trynda, Joanna Banach, Paweł Gretkierewicz, Joanna Wietrzyk

**Affiliations:** 1 Department of Experimental Oncology, Hirszfeld Institute of Immunology and Experimental Therapy, Polish Academy of Sciences, 53-114 Wroclaw, Poland; agata.pawlik@iitd.pan.wroc.pl (A.P.); artur.anisiewicz@iitd.pan.wroc.pl (A.A.); filip@iitd.pan.wroc.pl (B.F.-P.); turlej@iitd.pan.wroc.pl (E.T.); justynatrynda@wp.pl (J.T.); joanna.banach@iitd.pan.wroc.pl (J.B.); pgretkierewicz@gmail.com (P.G.); 2 Faculty of Veterinary Medicine, Wroclaw University of Environmental and Life Sciences, 50-3754 Wroclaw, Poland; marcin.nowak@upwr.edu.pl

**Keywords:** calcitriol, vitamin D analogs, breast cancer, metastasis, immunosuppression, osteopontin, TGF-β

## Abstract

In our previous study, calcitriol and its analogs PRI-2191 and PRI-2205 stimulated 4T1 mouse mammary gland cancer metastasis. Therefore, we aimed to analyze the inflammatory response in 4T1-bearing mice treated with these compounds. Gene expression analysis of the splenocytes and regional lymph nodes demonstrated prevalence of the T helper lymphocytes (Th2) response with an increased activity of regulatory T (Treg) lymphocytes in mice treated with these compounds. We also observed an increased number of mature granulocytes and B lymphocytes and a decreased number of TCD4^+^, TCD4^+^CD25^+^, and TCD8^+^, as well as natural killer (NK) CD335^+^, cells in the blood of mice treated with calcitriol and its analogs. Among the splenocytes, we observed a significant decrease in NK CD335^+^ cells and an increase in TCD8^+^ cells. Calcitriol and its analogs decreased the levels of interleukin (IL)-1β and IL-10 and increased the level of interferon gamma (IFN-γ) in the plasma. In the tumor tissue, they caused an increase in the level of IL-10. Gene expression analysis of lung tissue demonstrated an increased level of osteopontin (*Spp1*) and transforming growth factor β (TGF-β) mRNA. The expression of *Spp1* was also elevated in lymph nodes. Calcitriol and its analogs caused prevalence of tumor-conducive changes in the immune system of 4T1 tumor-bearing mice, despite the induction of some tumor-disadvantageous effects.

## 1. Introduction

Metastasis is a complex and multistage process of primary malignant tumor cells spreading to the secondary organs. To invade the surrounding stroma and disrupt the vascular endothelium, tumor cells initially acquire an invasive phenotype. Within the blood stream, the tumor cells must survive and evade physical damage, as well as evade attacks from the immune system [1,2]. Moreover, malignant cells interact with various stromal host cells at primary and secondary tumor locations. Prolonged inflammation is generally accepted as a potentiating factor in the progression of primary tumor and in metastasis [3,4,5,6].

Calcitriol, (1,25-dihydroxyvitamin D_3_; 1,25(OH)_2_D_3_), is a hormonally active vitamin D_3_ metabolite and an essential factor in maintaining the normal functioning of the immune system [7]. This is because of the fact that almost all immune cells express vitamin D receptor (VDR) and calcitriol is able to modulate its functions [8]. For example, the *Vdr* mRNA is expressed in subsets of the T helper lymphocytes (Th). Although Th1 or naïve T cells express low levels of *Vdr*, in differentiated Th17 and Th2 cells, and to a lesser extent in regulatory T (Treg) cells, the expression of *Vdr* has been found to be increased [9]. In addition, cells of the immune system with the 1-hydroxylase enzyme encoded by *CYP27B1* can generate calcitriol independently, regardless of the normal *CYP27B1* regulation in kidney via parathormone (PTH) [10,11]. Although many of the studies have focused on analyzing the effect of vitamin D on the immune system using in vitro and in vivo methods, there are only a few reports on the effect of vitamin D on the immune system during progression and metastasis of solid tumors. It is important to investigate the effects of vitamin D on immune response in solid tumors in the light of the evidence that calcitriol shows an immunosuppressive effect, which is conflicting with the expected properties of an effective anticancer agent in the case of solid tumors [12]. Therefore, researchers are proposing the selection of clinical indications in which systemic immunomodulatory effects of calcitriol could be minimized during treatment with vitamin D and its analogs, for example, in case of superficial bladder cancer [12]. Intravesicular combined treatment of bladder cancer with bacillus Calmette–Guerin (BCG) and calcitriol improved the therapeutic efficacy of the anticancer immunotherapeutic agent, increasing the production of interleukin (IL)-8, thereby enhancing the innate immune cell recruitment [13]. However, Guo et al., using the model of hepatocellular carcinoma, have shown that by increasing the cyclin dependent kinase inhibitor *p27^kip1^*, calcitriol reduces the secretion of pro-inflammatory cytokines and consequently inhibits signal transducer and activator of transcription 3 (STAT3) signaling activation, which eventually suppresses tumor development in mice [14]. Recently, in the model of the 4T1 mouse mammary gland cancer transplanted subcutaneously, Cao et al. have shown, that vitamin D stimulated the growth of primary tumors and decreased survival time of mice. The authors correlated this unfavorable effect with decreased Th1 response and increased recruitment of myeloid-derived suppressor cells [15].

Calcitriol, as well as other VDR agonists, are well-known inducers of apoptosis and cell cycle arrest and are inhibitors of metastatic invasion of breast cancer cells, as determined by in vitro methods*.* Despite the expression of VDR in breast cancer cells, vitamin D deficiency is very common in patients with breast cancer. Some authors suggest that a “correction of vitamin D deficiency or provision of supplemental vitamin D in women living with breast cancer would be predicted to delay recurrence and extend survival” [16,17,18].

The vitamin D deficiency may promote breast cancer metastasis [19]. Furthermore, the antimetastatic effect of VDR agonists in immune-deficient mice has been previously observed [18]. These findings motivated us to evaluate the antimetastatic activity of calcitriol and its analogs using the 4T1 mouse mammary gland cancer model transplanted orthotopically into immunocompetent mice. Using this model, we performed a series of experiments and reported that calcitriol and its low-toxic analogs PRI-2191 [20] and PRI-2205 [21] stimulated metastasis in 4T1 tumor-bearing mice [22]. The increased metastatic potential of 4T1 cells was accompanied with increased perfusion of blood in tumor tissue. During our previous aforementioned investigations, one of the cytokines, the transforming growth factor-β (TGF-β), was found to be significantly elevated in the plasma of mice treated with vitamin D and its analogs [22]. Therefore, in this study, we aimed to analyze the general inflammatory response of the 4T1 tumor-bearing mice treated with calcitriol, PRI-2191, and PRI-2205 to evaluate the role of immunosuppression as one of the mechanisms of pro-metastatic action of vitamin D and its analogs.

## 2. Results

### 2.1. Phenotypical Analyses of Cells Harvested from Blood

#### Phenotype of Peripheral Blood Lymphocytes

**Figure 1:**
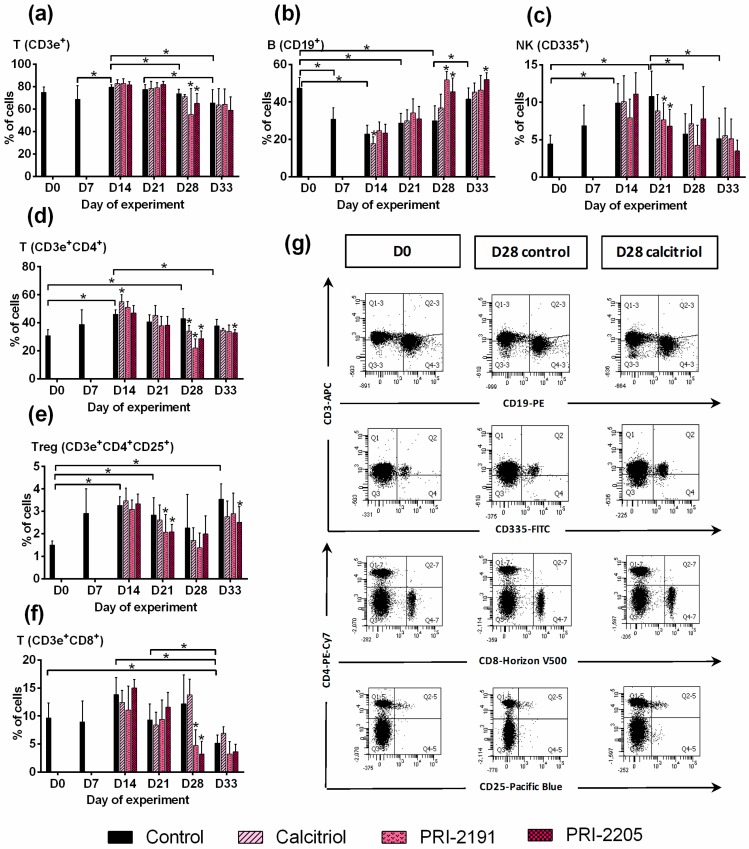
The phenotypes of peripheral blood lymphocytes in mice bearing 4T1 mammary gland tumors and treated with calcitriol, PRI-2191, and PRI-2205. (**a**) T lymphocytes CD3e^+^; (**b**) B lymphocytes CD19^+^; (**c**) natural killer (NK) cells CD335^+^; (**d**) TCD4^+^ lymphocytes; (**e**) TCD4^+^CD25^+^ lymphocytes; (**f**) TCD8^+^ lymphocytes; (**g**) representative dot plots of selected analysis performed on day 28. Data for calcitriol are shown as an example. The number of samples analyzed were 5–7 per group. Data were analyzed using the FACS Diva software. Data are presented as mean ± SD. Statistical analysis: Kruskal–Wallis multiple comparison test. * *p* < 0.05.

After treatment with calcitriol and its analogs, the CD4^+^ T lymphocytes were found to be increased on days 14 and 21 (on day 14, *p* < 0.05 for calcitriol; *p* = 0.0539 for PRI-2191). However, further treatment with study compounds caused a significant decrease in the population of CD4^+^ cells (Figure 1d). We further analyzed the subpopulation of the regulatory T cells (Tregs; CD25^+^). Beginning from day 21, the percentage of CD25^+^ cells was found to be decreased in mice treated with calcitriol and its analogs (*p* < 0.05 on days 21 and 28 for PRI-2191 and PRI-2205 or only for PRI-2205, respectively) (Figure 1e).

After treatment with the study compounds, we observed a significant decrease in the percentage of natural killer (NK) cells on day 21 and of T CD8^+^ cells on day 28 (Figure 1c,f).

On day 14, calcitriol (but not its analogs) significantly decreased the percentage of B cells. All compounds tended to increase the percentage of CD19^+^ cells on days 21, 28, and 33 (*p* < 0.05 on day 28 for both analogs and on day 33 for PRI-2205) (Figure 1b).

The blood morphological analysis is presented in the Supplementary Material (Figures S1 and S2).

### 2.2. Characteristics of Spleen Lymphocytes

#### 2.2.1. Phenotype RT² Profiler PCR Array (“Mouse T Helper Cell Differentiation”) Analysis of Gene Expression

**Figure 2:**
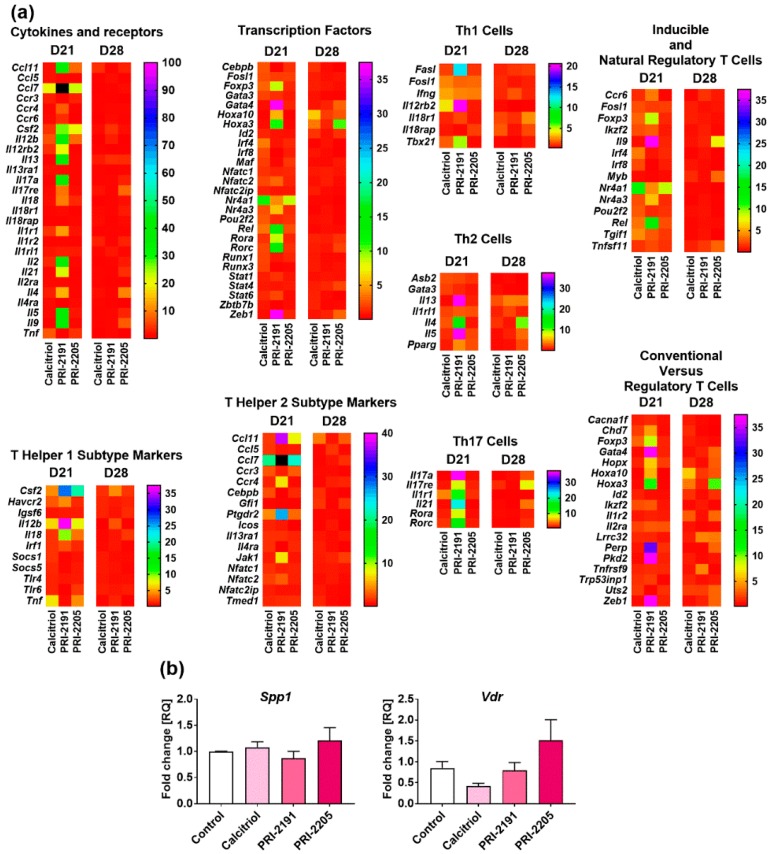
The gene expression profile in isolated mononuclear splenocytes from 4T1 tumor-bearing mice treated with calcitriol and its analogs. (**a**) Heat maps presenting mean relative quantification (RQ) values from duplicates. The genes were grouped according to the analysis provided by manufacturer: cytokines and receptors, transcription factors, T helper lymphocytes (Th1) and Th2 subtype markers, and epigenetically regulated genes: conventional versus regulatory T cells, inducible and natural regulatory T cells, Th1, Th2, and Th17 cells. Values outside the scale are marked in black. Detailed data can be found in the Table S1. Fold-change (RQ) of target cDNA was determined using ΔΔCt analysis with reference to beta-actin (*Actb*) and beta-2 microglobulin (*B2m*) and adjusted to the values obtained for the vehicle treated control mice for each treatment group; (**b**) the expression of vitamin D receptor (*Vdr*) and osteopontin (*Spp1*) mRNA levels (day 28). Fold-change (RQ) of target genes was determined using ΔΔCt analysis compared to beta-2 microglobulin (*B2m*) and adjusted to the values obtained for the vehicle treated control mice for each treatment group. Data presentation: mean with standard error of mean. Number of samples analyzed was 2–3 per group. Statistical analysis: Kruskal–Wallis multiple comparison test. * *p* < 0.05.

Calcitriol and its analogs significantly modified the expression of genes in splenocytes in our studies. Therefore, we decided to describe only those genes that were upregulated more than 10 times or that were downregulated more than 5 times (Figure 2a).

On day 21, expression of the following genes was found to be upregulated: genes related to Th1, such as colony stimulating factor 2 (granulocyte-macrophage) (*Csf2*) and interleukin 2 (*Il2*); and those related to Th2 cells, such as chemokine (C-C motif) ligand 7 (*Ccl7*), *Ccl11*, prostaglandin D2 receptor 2 (*Ptgdr2*), *Il13*, *Il4*, *Il5*, and *Il9*. Expression of the genes related to Treg cells was also found to be upregulated on day 21: homeobox A3 (*Hoxa3*) and reticuloendotheliosis oncogene, NF-κB subunit (*Rel*). On day 21, the following Th17-related genes were also upregulated (but not as much as the aforementioned genes): interleukin 17a (*Il17a*), interleukin 17 receptor E (*Il17re*), interleukin 1 receptor type 1 (*Il1r1*), *Il21*, RAR-related orphan receptor alpha (*Rora*), and RAR-related orphan receptor gamma (*Rorc*).

On the 28th day of the experiment, the interferon regulatory factors 4 and 8 (*Irf4* and *Irf8*), both related to Treg cells, were the most downregulated genes.

The following groups of genes were upregulated on day 21 and were downregulated on day 28, related to Th1 cells: interleukin 12b (*Il12b*), interleukin 12 receptor, beta 2 (*Il12rb2*), tumor necrosis factor (*Tnf*); related to Th2 cells: ankyrin repeat and SOCS box containing 2 (*Asb2*); and related to Treg cells: nuclear receptor subfamily 4 group A members 1 and 3 (*Nr4a1* and *Nr4a3*), transforming growth factor β (TGF-β)-induced factor homeobox 1 (*Tgif1*) (Figure 2a). On day 21, the expression of *Foxp3* mRNA was found to be upregulated by calcitriol (~3-fold), PRI-2191 (~9-fold), and PRI-2205 (~2-fold), whereas on day 28, calcitriol or its analogs did not change the expression of *Foxp3* (Figure 2a). Table S1 lists all fold change values.

#### 2.2.2. *Vdr* and *Spp1* mRNA Level in Splenocytes

Calcitriol downregulated and PRI-2205 upregulated the expression of *Vdr*. However, there were no statistically significant changes (Figure 2b).

#### 2.2.3. Phenotype of Spleen Lymphocytes

**Figure 3:**
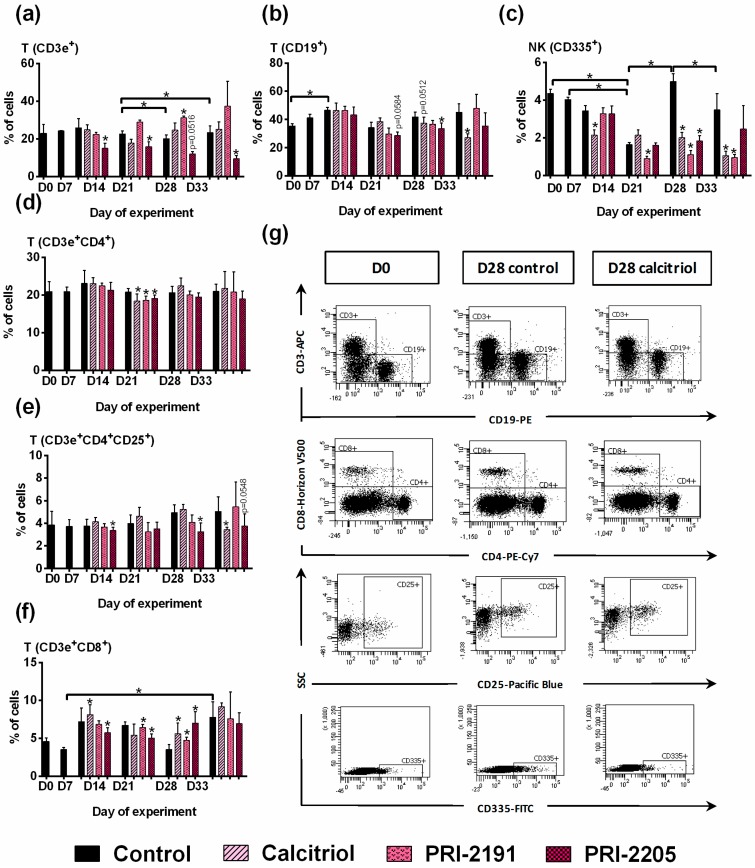
Spleen lymphocyte phenotypes in mice bearing 4T1 mammary gland tumors treated with calcitriol, PRI-2191, and PRI-2205. (**a**) T lymphocytes CD3e^+^; (**b**) B lymphocytes CD19^+^; (**c**) NK cells CD335^+^; (**d**) TCD4^+^ lymphocytes; (**e**) TCD4^+^CD25^+^ lymphocytes; (**f**) TCD8^+^ lymphocytes; (**g**) representative dot plots of selected analysis performed on day 28. Data for calcitriol are shown as an example. Number of samples analyzed was six per group with the following exception: D0 = 2 and D7 = 3. Data were analyzed using the FACS Diva software. Data are presented as mean ± SD. Statistical analysis: Kruskal–Wallis multiple comparison test. * *p* < 0.05.

From day 14 to 33, PRI-2205 decreased the percentage of T lymphocytes. Calcitriol, and especially PRI-2191, tended to increase the percentage of these cells (Figure 3a). Among the T lymphocytes, the percentage of CD4^+^ cells was found to be decreased on day 21 after treatment (Figure 3d). Calcitriol and PRI-2205 decreased the percentage of CD4^+^CD25^+^ cells significantly on day 33 (Figure 3e). All tested compounds decreased the percentage of NK CD335^+^, which was found to be greater on days 28 and 33 (Figure 3c). A significant increase in CD8^+^ cells was observed in mice treated with all compounds, especially on day 28 (Figure 3f).

### 2.3. Cytokine Analysis in 4T1 Tumor-Bearing Mice Treated with Calcitriol, PRI-2191, and PRI-2205

#### 2.3.1. Cytokine Arrays Analysis of Plasma and Supernatants from Lipopolysaccharide (LPS)- or Concanavalin A (ConA)-Stimulated Splenocytes

Plasma cytokines or cytokines secreted into the culture medium by stimulated splenocytes were screened. Spleens were harvested from healthy mice (indicated as D0) and from tumor-bearing mice on days 7, 21, and 33 after transplantation with cancer cells (D7).

**Figure 4:**
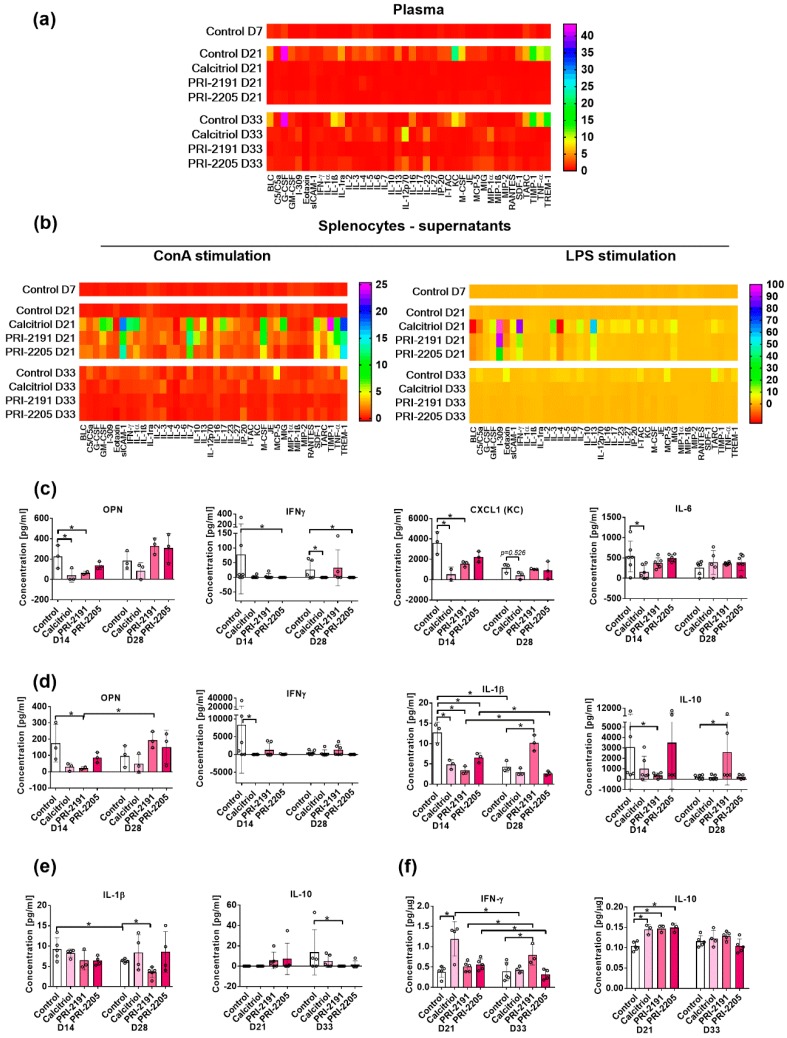
Analysis of cytokine arrays of plasma or supernatants from lipopolysaccharide (LPS)-/Concanavalin A (ConA)-stimulated splenocytes and expression of selected cytokines in tumor tissue. (**a**,**e**) Plasma; (**b**–**d**) supernatants obtained from spleen cells stimulated with LPS and ConA; (**f**) tumor tissue lysates. (**a**,**b**)—samples analyzed with proteome profiler array. Samples from three randomly selected mice were pooled in each tested group. Data are presented as fold change as compared with healthy (D0) mice samples; (**c**,**d**) supernatants obtained from spleen cells (harvested on days 14 and 28) stimulated with (**c**) LPS and (**d**) ConA; (**e**) plasma from mice harvested on days 14 or 21 and 28 or 33; (**f**) tumors harvested on days 21 and 33, homogenized and lysed. (**c**–**f**) samples were analyzed with ELISA tests. Number of samples analyzed were 2–6 per group. Data are presented as mean ± SD and individual sample results. Statistical analysis: Kruskal–Wallis multiple comparison test. * *p* < 0.05. OPN—osteopontin; IFN-γ—interferon gamma; IL—interleukin; CXCL-1—chemokine (C–X–C motif) ligand 1; G-CSF—granulocyte-colony stimulating factor; GM—granulocyte-macrophage; sICAM-1—soluble intercellular adhesion molecule-1; M-CSF—macrophage colony-stimulating factor; MCP-5—monocyte chemotactic protein 5; MIG—macrophage-induced gene; MIP—macrophage inflammatory protein; SDF-1—stromal cell-derived factor 1; TIMP-1—stromal cell-derived factor 1; TNF—tumor necrosis factor; TREM—triggering receptor expressed on myeloid cells.

On day 21, almost all cytokines were found to be downregulated in plasma by calcitriol and its analogs. However, on day 33, some of the cytokines were found to be upregulated, such as eotaxin, IL-1α, IL-10, IL-13, IL-12p70, IL-23, monocyte chemotactic protein 5 (MCP-5), macrophage-induced gene (MIG), and macrophage inflammatory protein (MIP)-1α (Figure 4a and Table S2A–E).

Calcitriol and its analogs modulated the levels of cytokines secreted from LPS-stimulated splenocytes. On day 21, granulocyte-colony stimulating factor (G-CSF), granulocyte-macrophage (GM)-CSF, IL-4, IL-6, and IL-10 were found to be increased by calcitriol and its analogs, whereas on day 33, their level was found to be decreased as compared with control tumor-bearing mice. However, IL-2 was found to be decreased on both days 21 and 33 by calcitriol and its analogs (Figure 4b and Table S3A–E).

In ConA-stimulated splenocytes (day 21), calcitriol and its analogs stimulated the secretion of GM-CSF, soluble intercellular adhesion molecule-1 (sICAM-1), IFN-γ, IL-1α and β, IL-3, IL-4, IL-7, IL-17, IL-27, macrophage (M)-CSF, stromal cell-derived factor 1 (SDF-1), tissue inhibitor of metalloproteinases (TIMP)-1, tumor necrosis factor (TNFα), and triggering receptor expressed on myeloid cells (TREM)-1 into the culture media. The secretion of IL-12p70, MIP-1β, and MIP-2 was downregulated by all study compounds, whereas other cytokines remain unchanged. On day 33, almost all cytokines were found to be decreased in ConA-stimulated splenocytes in case of mice treated with calcitriol and its analogs (Figure 4b and Table S4A–E).

#### 2.3.2. Plasma Levels of Selected Cytokines Measured by ELISA Test

The plasma concentration of IL-1β was found to be decreased by calcitriol and its analogs, especially by PRI-2191 (*p* < 0.05 on day 28). The plasma concentration of IL-10 in control mice was found to be elevated from a non-detectable level on day 21 to a detectable level on day 33 in three out of five mice. All study compounds tended to decrease the level of IL-10 on day 33 (*p* < 0.05 for PRI-2191-treated mice), whereas both analogs tended to increase its level on day 21 (Figure 4e). On days 21 and 28, IL-2 and IL-4 were found to be undetectable in almost all mice with the exception of control animals on day 28 (IL-2) and day 21 (IL-4). Calcitriol and its analogs did not significantly affect the plasma level of IFN-γ and IL-6 (Figure S3C).

#### 2.3.3. Levels of Selected Cytokines in Supernatants from Stimulated Splenocytes

LPS- or ConA-stimulated splenocytes produced low level of TGF-β (Figure S3). CXCL-1 chemokine secretion was inhibited by calcitriol and its analogs in LPS- and ConA-stimulated splenocytes (Figure 4c,d). The level of OPN in the supernatants was found to be decreased by calcitriol and its analogs (both in LPS- and ConA-stimulated splenocytes harvested from mice on day 14). On day 28, only calcitriol tended to decrease the level of osteopontin (OPN), but both analogs tended to increase its level (Figure 4c,d). IL-1β secretion was found to be decreased in LPS- and ConA-stimulated splenocytes collected on day 14 (*p* < 0.05), whereas in mice with more advanced tumors (day 28), the secretion of IL-1β did not change in mice treated with calcitriol or PRI-2205 as compared with control mice, but was found to be increased by PRI-2191 (Figure 4d and Figure S3A). IL-2 and IL-4 were not found to be significantly affected by calcitriol and its analogs (Figure S3A,B). The level of IL-6 from LPS-stimulated splenocytes remained unchanged by PRI-2191 and PRI-2205; however, calcitriol decreased its level on day 14 (*p* < 0.05). Calcitriol showed a similar tendency of decreasing the level of IL-6 on day 14 after ConA stimulation (Figure 4c and Figure S3B). On day 14, the level of IL-10 was found to be decreased by calcitriol and its analogs (*p* < 0.05 for PRI-2191 ConA stimulation). On day 28, PRI-2191 significantly increased the secretion of this cytokine in ConA-stimulated splenocytes (Figure 4d and Figure S3A). In ConA-stimulated splenocytes, IFN-γ secretion diminished in mice treated with calcitriol and its analog, especially on day 14, but in LPS-stimulated splenocytes, the same tendency was observed on day 28 (Figure 4c,d).

### 2.4. Immunological Response in Lymph Nodes during 4T1 Tumor Progression in Mice Treated with Calcitriol, PRI-2191, and PRI-2205

#### RT² Profiler PCR Array (“Mouse T Helper Cell Differentiation”) Analysis of Gene Expression

Calcitriol and its analogs upregulated the expression of some genes in lymph nodes, such as genes related to Th2 cells by two- to eight-fold: ankyrin repeat and SOCS box containing 2 (*Asb2*), chemokine (C-C motif) ligand 7 (*Ccl7*), CCAAT/enhancer binding protein (C/EBP), beta (*Cebpb*), *Il13*, and *Il4*. The expression of the following genes related to Treg cells was also elevated: calcium voltage-gated channel subunit alpha1 F (*Cacna1f*), FOS like 1 (*Fosl*), GATA binding protein 4 (*Gata4*), urotensin 2 (*Uts2*), and zinc finger E-box binding homeobox 1 (*Zeb1*). An approximately two-fold increase in the expression of *Foxp3* was also observed.

**Figure 5:**
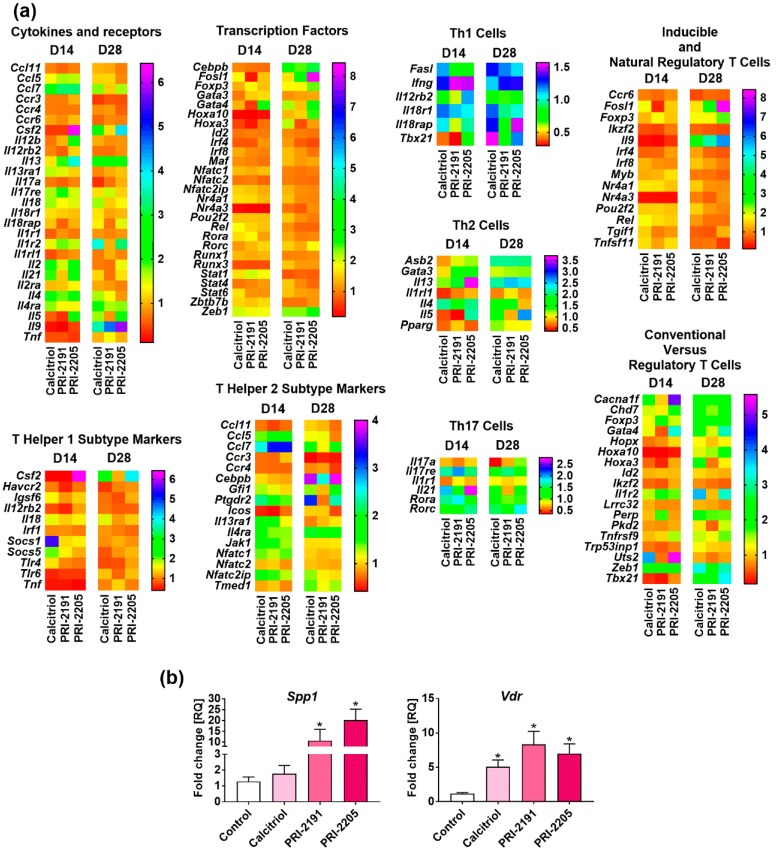
The gene expression in regional lymph nodes from 4T1 tumor bearing mice treated with calcitriol and its analogs. (**a**) Heat maps presenting mean relative quantification (RQ) values from duplicates. The genes were grouped according to the analysis provided by the manufacturer: cytokines and receptors, transcription factors, Th1 and Th2 subtype markers, and epigenetically regulated genes: conventional versus regulatory T cells, inducible and natural regulatory T cells, Th1, Th2, and Th17 cells. Lymph node specimens were collected on the days 21 and 28 (after inoculation with 4T1 cells). Fold-change (RQ) of target cDNA was determined using ΔΔCt analysis with reference to actin, beta (*Actb*), and beta-2 microglobulin (*B2m*) and adjusted to the values obtained for the vehicle treated control mice for each treatment group; (**b**) the expression of vitamin D receptor (*Vdr*) and osteopontin (*Spp1*) mRNA levels (day 28). Data presentation: mean with standard error of mean. Fold-change (RQ) of target genes was determined using ΔΔCt method compared with beta-2 microglobulin (*B2m*) and adjusted to the values obtained for the vehicle treated control mice for each treatment group. Number of samples analyzed were 4–6 per group. Statistical analysis: Kruskal–Wallis multiple comparison test. * *p* < 0.05.

On day 14, we identified some of the genes that were downregulated by calcitriol and its analogs. The following genes related to Th1 cells were downregulated: tumor necrosis factor (*Tnf*) and T-box 21 (*Tbx21*), and those related to Treg cells, such as nuclear receptor subfamily 4 group A member 3 (*Nr4a3*). Moreover, a single gene, chemokine (C–C motif) receptor 3 (*Ccr3*), was found to be downregulated only on day 28 (related to Th2). On days 14 and 28, a single gene, *Il17a*, was found to be downregulated by calcitriol and its analogs (related to Th17). *Hoxa10*, *Csf2*, and *Il9* were found to be downregulated on day 14 and upregulated on day 28 (Figure 5a). Table S5 shows all fold change values.

### 2.5. Vitamin D Receptor (Vdr) and Osteopontin (Spp1) mRNA Levels in Lymph Nodes

We found a statistically significant increase of *Vdr* in mice treated with all the study compounds. PRI-2191 and PRI-2205 significantly increased the level of *Spp1* in lymph nodes. Calcitriol showed a similar tendency, but the increase was not significant (Figure 5b).

### 2.6. Immune Response of Tumor and Lung Tissue from 4T1 Mammary Gland Tumor-Bearing Mice Treated with Calcitriol, PRI-2191, and PRI-2205

#### 2.6.1. Level of Selected Cytokines in Tumor Tissue

Calcitriol (day 21) and PRI-2191 (day 33) significantly increased the level of INF-γ (Figure 4f). All study compounds decreased the level of IL-1β on day 28, but only PRI-2191 decreased its level significantly (Figure S4a). IL-5 and IL-6 were not found to be affected significantly by the study compounds (Figure S4b,c). However, on day 21, IL-10 was found to be significantly elevated by calcitriol and its analogs (Figure 4g).

#### 2.6.2. Gene Expression Profile in Lung Tissue

**Figure 6:**
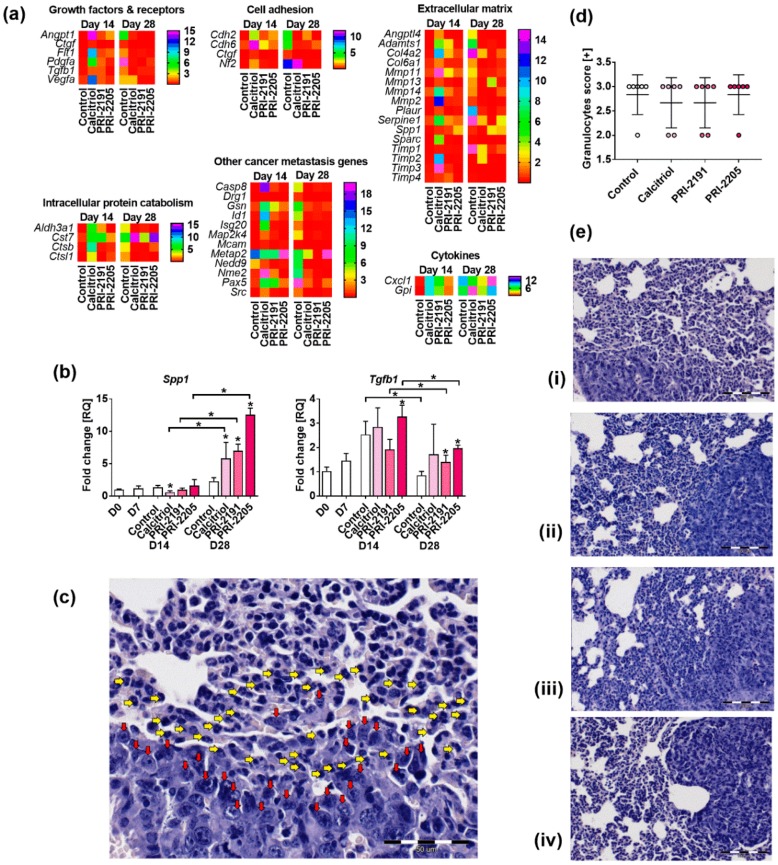
Changes in mRNA expression and granulocyte infiltration in lung from mice treated with calcitriol and its analogs. (**a**) Screening of genes correlated with tumor invasion and metastasis using real-time PCR; (**b**) real-time PCR analysis of two selected genes; (**c**–**e**) lung infiltration by granulocytes: (**c**) representative picture of lung tissue with metastatic focus (magnification of 400×, scale bar 50 µm) infiltrated by granulocytes. On the margin of tumor tissue: red arrows—nuclei of tumor cells, yellow arrows—nuclei of granulocytes; (**d**) scoring of granulocytes infiltration in lung tissue; (**e**) representative pictures of lung tissue sections (magnification of 200×, scale bar 100 µm): (**i**) control, (**ii**) calcitriol, (**iii**) PRI-2191, and (**iv**) PRI-2205. Number of mice evaluated were 3–7 per group. Data presentation: (**a**) heat maps presenting mean relative quantification (RQ) values from duplicates. Fold-change (RQ) of target cDNA was determined by calculating the differences in ΔΔCT values with reference to ribosomal protein L13A (*Rpl13a*) and adjusted to the values obtained for the untreated mice (named D0) for each treatment group; (**b**,**d**) mean ± SD. Statistical analysis: Kruskal–Wallis multiple comparison test, * *p* < 0.05.

Calcitriol and its analogs upregulated the expression of the following genes on day 14 and downregulated their expression on day 28: angiopoietin 1 (*Angpt1*), vascular endothelial growth factor receptor 1 (*Flt1*), transforming growth factor β1 (*Tgfb1*), connective tissue growth factor (*Ctgf*), vascular endothelial growth factor A *(Vegfa*), and platelet-derived growth factor α (*Pdgfa*) (Figure 6a). Calcitriol and PRI-2191 showed a similar profile in the regulation of gene expression for most of the analyzed mRNAs. PRI-2205 also showed a similar tendency, but not as clear as the other two compounds. The expression of some genes differed from the above mentioned pattern. For example, upregulation of the following mRNA levels was observed on both days of analysis (day 14 and 28): cystatin F (leukocystatin; *Cst7*), glucose phosphate isomerase (*Gpi*), and secreted phosphoprotein 1 (osteopontin (OPN), *Spp1*). Moreover, only calcitriol upregulated neurofibromin 2 (merlin; *Nf2*) and TIMP metallopeptidase inhibitor 2 (*Timp2*), whereas only PRI-2205 upregulated methionyl aminopeptidase 2 (*Metap2*) expression (Figure 6a and Table S6).

We performed a real-time PCR analysis of *Spp1* and *Tgfb* in samples of lung tissue. The expression of *Spp1* and *Tgfb* was found to be significantly upregulated on day 28 by study compounds (Figure 6b). On day 14, calcitriol decreased the expression of *Spp1*, whereas PRI-2205 increased the expression of *Tgfb1* (Figure 6b).

#### 2.6.3. Granulocytes Lung Tissue Infiltration

Granulocytes were found to infiltrate the lung tissue of 4T1-tumor bearing mice. Figure 6c shows a photograph of a lung tissue with an evident metastatic lesion (higher magnification of the tissue from control mice: Figure 6e(i)). On the border of the metastatic foci, the nuclei of the cancer cells have been marked with red arrows and the nuclei of the granulocytes with yellow arrows. Regardless of the treatment, infiltration of granulocytes was similar in mice in all groups (Figure 6d,e).

## 3. Discussion

The hormonally active form of vitamin D_3_ is known to regulate calcium and phosphorus homeostasis. However, it might also cause toxicity because of the application of potentially active, hyper-physiological doses in cancer treatment, which motivated the researchers to synthesize new analogs, with a split calcemic and antiproliferative activity [23]. One of such compounds synthesized is the vitamin D_3_ metabolite, (24*R*)-1,24-dihydroxyvitamin D_3_ (tacalcitol, 1,24(OH)_2_D_3_, PRI-2191). It is well documented that PRI-2191 inhibits cancer cell proliferation by inducing the differentiation of epidermal mouse and human keratinocytes in the case of psoriasis, and leukemic cells in the case of acute myeloid leukemia [24,25]. PRI-2191 also binds to the VDR with an affinity greater than or similar to that of calcitriol, but shows weaker induction of calcemia than that of calcitriol [20,26]. The analogs of vitamin D with a reversed (5*E*,7*E*) geometry of the triene system have been previously reported by our team [21,27] and by another group [28] to show the enhanced antitumor activity as compared with the natural (5*Z*,7*E*) vitamin D compounds. An example of such analog is the PRI-2205, deprived of calcemic activity [21]. The mechanism of action of PRI-2191 in cancer cells has been shown to be similar to calcitriol and is related to the induction of cancer cell differentiation, for example, by increasing the expression of E-cadherin in colon cancer cells [26,29]. However, the mechanism of action of PRI-2205 seems to be different; it induces apoptosis in leukemic cells [21] and enhances the antitumor and antimetastatic activity of 5-fluorouracil (5-FU) and capecitabine in colon cancer in vivo via mechanisms that are not related to the process of cell differentiation [29]. In our previous study, PRI-2205, similar to calcitriol and PRI-2191, induced metastatic spread of the 4T1 mammary gland cancer transplanted orthotopically (the number of metastases increased by 58%, 50%, and 54% over control, respectively, on day 33, Table S7) [22], but the activity of PRI-2205 toward immune response presented herein seems to be different in some aspects. Therefore, in the subsequent paragraphs, we commonly discuss the results obtained for calcitriol, PRI-2191, and PRI-2205, only when the direction of their activity is the same, without analysis of different results for PRI-2205.

According to the literature, calcitriol and its analogs slow down the growth of human [30,31,32] or mouse [32,33] breast cancer. Moreover, a vitamin D-deficient diet accelerated tumor growth [19] and bone metastasis [34] of transplanted mouse MMTV*-Wnt1* or human MDA-MB-231 breast cancer cells, respectively. However, the exogenous 25-hydroxyvitamin D (25(OH)D) delays spontaneous mammary gland neoplasia [35]. In our previous studies using the model of human and mouse colon cancer, we observed that PRI-2191, when used with 5-fluorouracil, decreased primary tumor growth and the number of lymph node metastases [26,29]. Furthermore, we have previously observed decreased tumor growth in the lung A549 xenograft when PRI-2191 was combined with cytostatic and tyrosine kinases inhibitors [36,37]. However, the cancer cells used in the aforementioned studies, such as MDA-MB-231 [34], MCF-7 [32], MMTV-*PyMT* [35], MMTV-*Wnt1* [19], and HT-29 [26], were sensitive to the direct action of vitamin D and/or immune deficient models were used [26,34,36,37]. Although 4T1 cells express VDR, they are not sensitive to calcitriol in vitro nor in vivo; cell proliferation and primary tumor growth was not affected upon the treatment [22]. Zhang et al. reported contrasting results compared with ours using the same experimental model (4T1); they reported a reduction in the number of lung metastases in 4T1-tumor bearing mice after treatment with calcitriol [38]. On the other hand, Cao et al., using 4T1 cells transplantated subcutaneously (s.c.), report on stimulation of primary tumor growth and reduced survival of mice upon vitamin D treatment. It seems that such divergent observations may be the result of a different scheme of the experiment. For example, Zhang et al. administered calcitriol intraperitoneally one day prior to the inoculation of cancer cells. In case of our study or the one conducted by Cao et al., administration of calcitriol was started 7 or 17 days, respectively, after mice inoculation with the 4T1 cells. However, similar to our results, Zhang et al. also did not notice any significant influence of calcitriol on in situ tumor growth. In our previous studies, we have shown that PRI-2205 did not affect the growth of the primary tumor. However, it increased the number of lung metastases in the 4T1 model, whereas in combination with cyclophosphamide, it significantly increased its anti-cancer properties [39].

Based on our research and the results of other aforementioned studies, we can conclude that it is important to study the effect of calcitriol and its analogs, not only in mice bearing cancers that are sensitive to direct action of these compounds, but also in non-sensitive tumors, where the influence of these compounds may play an important role on the entire host organism, including tumor stroma and the immune system. Interestingly, the modulating role of calcitriol and its analogs may play different roles at different stages of tumor progression. Thus, we can assume that the use of calcitriol or its analogs before tumor implantation [38] or after the administration of immunosuppressive cytostatic cyclophosphamide [39]—therefore, in the body in which the inflammatory process accompanying the development of 4T1 did not start or was suppressed—will have a different effect than the results reported here. The results of this study have shown that calcitriol and its analogs stimulated inflammatory response (increased mRNA level of acute phase proteins in the liver, Figure S5), which is characteristic during the progression of 4T1 tumor [40]. In addition, 4T1 tumor development is accompanied by gradually progressing leukocytosis correlated with splenomegalia [40,41]. Calcitriol and its analogs increased the percentage of lymphocytes and monocytes, whereas they decreased the percentage of granulocytes (Figure S1). This encouraged us to conduct further analysis with respect to the immune modulation in 4T1 tumor-bearing mice after the administration of calcitriol and its analogs.

To evaluate the local and whole body immune response in 4T1 tumor-bearing mice after administration of calcitriol and its analogs, we performed the selected mRNA profiling of splenocytes and regional lymph nodes, as well as cytokine arrays of plasma and ex vivo*-*stimulated splenocytes. The most upregulated genes by calcitriol and its analogs were the typical ones or those related to Th2 and Treg cells. It is known that these cells are engaged in tumor-conducive immunosuppressive response [42]. In particular, in splenocytes and/or lymph nodes, we have observed an increased expression of mRNA of Th2 signature cytokines [43], such as *Il4*, *Il5*, *Il9*, or *Il13*. However, in splenocytes alone, calcitriol increased the level of *Tnf*, *Il12b*, and *Il12rb2* at an early stage of tumor progression and decreased their levels during the advanced phase of the experiment. Moreover, calcitriol stably increased the expression of *Il2* in splenocytes. The Treg development from naïve periphery CD4^+^ T lymphocytes (pTreg) is triggered by the combination of cytokines, namely IL-2 and TGF-β (in addition to the antigen stimulation), but signaling of both cytokines is important in pTreg, as well as thymus derived Treg (tTreg), differentiation [44]. In the splenocytes, this activity of calcitriol and its analogs lead to the upregulation of *Rel*, an important gene in Treg differentiation. It is one of the subunits of NF-ĸB, and it has been shown to be essential in the differentiation and activation of Treg, as well as during cancer progression [45]. In addition, it has been demonstrated that c-Rel (protein encoded by *Rel*) is responsible for the development of tTreg cells, but not for peripherally induced pTregs [46,47]. Other transcription factors responsible for the development of tTregs are members of the NR4a family [44]. Interestingly, our results have shown that mRNA levels of both *Nr4a1* and *Nr4a3* were increased in spleen during the early stage of tumor progression and were decreased during advanced stages. These transcription factors are known as strong inducers of *Foxp3* [48]. In our studies, the expression of *Foxp3* was found to be elevated (up to 8-fold) in splenocytes from mice treated with calcitriol and its analogs, but only during the early stage of tumor growth (day 21). On day 28, the expression level of *Foxp3* was found to be similar to control tumor-bearing mice. Interestingly, transient increase in the levels of *Il12b* and *Il12rb2* in splenocytes from mice treated with calcitriol and its analogs can contribute to the lack of effect on the *Foxp3* expression level during the later stage of tumor progression. The IL-12, with its receptors, can participate in the conversion of Tregs into the Th1-like cells, as well as in maintaining Th1/Th2 hybrids. This process is known as the T cells plasticity [49,50]. However, the metastatic dissemination process in 4T1 tumor-bearing mice started about 1–2 weeks after the transplantation of tumor cells [41] and immunosuppressive cells can promote metastasis during all stages of metastatic cascade [51]. As it has been proven, cancer immunoediting is a dynamic process (consisting of the following phases: elimination, equilibrium, and escape) and during particular stages of carcinogenesis, the immune system can either destroy or promote tumor growth [52]. Therefore, different response of the immune cells to calcitriol and its analogs at the beginning and at the end of the experiment may be a reflection of the influence of growing tumors (not inhibited by the treatment) altering the inflammatory response.

Previous studies have revealed that the secretion of IFN-γ and IL-2 by T cells decreases during exposure to calcitriol, whereas the production of IL-4, IL-5, and IL-10 increases, thereby resulting in a shift toward Th2 cells’ response [53,54,55]. Although our gene profiling studies clearly indicated the shift in the immune response toward tumor promotion, mainly at earlier stages of the tumor progression, the cytokine profile in plasma have shown the downregulation of almost all cytokines by calcitriol and its analogs. However, on day 21, the stimulated splenocytes from mice treated with calcitriol and its analogs produced higher levels of cytokines. On day 33, the production of these cytokines was found to be decreased compared with control 4T1 tumor-bearing mice. The influence of calcitriol toward the secretion of cytokines, for example IL-10, may differ in various experimental conditions. Several studies have shown that the expression of IL-10 is induced by calcitriol in various immune cells, and in this way, the known immunosuppressive effect of calcitriol is mediated [54,56]. However, the inhibitory effect of calcitriol on the IL-10 production in vitro, as well as in vivo, has also been reported [57]. This contradictory result might be due to the different cell types studied. Moreover, we hypothesize that the upregulation of IL-10 is related to the duration of the treatment with calcitriol. Indeed, the treatment with calcitriol for several days consistently showed an upregulation of IL-10, whereas there was no regulation or inhibition of IL-10 during shorter incubation times used [58]. In this study, we observed a downregulation of IL-10 secretion by splenocytes during the early stages of tumor progression (day 14), which subsequently increased. In addition, in tumor tissue, IL-10 was found to be upregulated by calcitriol and its analogs during the early stages of tumor growth. Moreover, when we calculated the IL-10/IL-12p70 ratio, which can represent the balance between Th1/Th2 responses [59], it turned out that on day 33, splenocytes from mice treated with calcitriol or its analogs, and stimulated with LPS, produced 18–21 times higher levels of IL-10 over IL-12p17. Although the elevated level of IL-10 over IL-12 suggests the predomination of Th2 response during the treatment, further studies are necessary to exactly determine the type of cells that adopts such a behavior in response to treatment with calcitriol and its analogs during tumor progression.

The growth of 4T1 cells is accompanied by the elevated secretion of cytokines such as G-CSF and chemokine (C–X–C motif) ligand 1 (CXCL1, KC) [40,41], but calcitriol and its analogs only significantly diminished CXCL1 during the early stage of tumor progression, not affecting the secretion of G-CSF in a significant manner. High amounts of G-CSF, the cytokine that is able to switch the T cell cytokine secretion profile to Th2 responses, promote the regulatory T cell and modulate cytokine production [60], and may veil the activity of calcitriol in terms of secretion of various other cytokines. Moreover, the G-CSF enhances proliferation and mobilization of Ly6G^+^Ly6C^+^ granulocytes and facilitates their homing during metastasis of the target organs even before the onset of cancer cells, which facilitate metastatic process [61]. Unfortunately, calcitriol and its analogs increased the percent of Ly6G-6C^+^SCC^high^ cells (with a parallel decrease in the percentage of activated CD54^+^ (I-CAM) and CD184^+^ (CXCR4) granulocytes, Figure S6) in the blood, which correlated with an increased metastatic potential [22]. The Ly6G-6C^+^SCC^high^ granulocytes may serve as the primary source of TGF-β [62], which was found to be increased in plasma of 4T1 tumor-bearing mice administered with calcitriol and its analogs in our previous study [22]. The increased percentage of mature granulocytes and the production of cytokines such as TGF-β from these cells consequent to the treatment with calcitriol and its analogs may be responsible for lung metastatic niche formation and the enhancement of metastasis of 4T1 cells [61,63].

Another cytokine that was found to be elevated as a result of calcitriol treatment in 4T1 tumor tissue was OPN [22]. OPN is known to contain vitamin D response element (VDRE) in the promoter region of *Spp1* and calcitriol stimulates various cells to secrete OPN [64,65,66]. OPN stimulates tumor growth and promotes metastasis by influencing tumor angiogenesis [67,68,69]. Unexpectedly, we found that the stimulated splenocytes from calcitriol- or PRI-2191-treated mice secreted lower levels of OPN than that from control mice, especially during the early stage of the tumor progression. We did not find any literature data indicating inhibitory effects of calcitriol on OPN secretion. One of the possible explanations may be the decreased number of activated T and B cells upon calcitriol treatment, which has been described in the literature [70,71], and only activated T and B cells can produce OPN [72]. The elevated level of OPN in tumor tissue, observed in our previous studies upon treatment with calcitriol and its analogs [22], might also be the result of fibroblast stimulation, which is the major source of OPN in tumor tissue [73,74]. The almost unchanged plasma level of OPN in treated mice [22] is the result of applying such opposing effects of treatment. However, further studies should be performed to explain the effect of calcitriol on the secretion of OPN by T and B lymphocytes and other cells of the immune system in tumor-bearing organisms.

In the OPN knockout mice (*Spp1−/−*), the decreased frequency of Treg cells was found to be related with increased CD4^+^-activated T cells (CD44^+^CD69^+^ and CD62L^low^CD69^+^ cells) in metastatic niche (lungs), indicating OPN as the cytokine responsible for drawing the immunosuppression in the microenvironment of the metastatic niche [69]. In addition, TGF-β itself has a significant effect on CD4^+^ T-cells. The TGF-β induces the generation of Foxp3 and Treg cells [75]. TGF-β also inhibits the proliferation and functioning of NK-cells. The NK-cells are modulated in part by CD4^+^CD25^+^ regulatory T cells that are known from TGF-β high level production [75]. Our results have shown that the number of CD4^+^ T cells, CD4^+^CD25^+^ regulatory T cells, CD335^+^ NK, and CD8^+^ cells diminished in blood after treatment with calcitriol and its analogs. However, in the spleen, only NK and CD4^+^ cells were found to be decreased, whereas CD8^+^ cells were found to be increased, which was clearly visible on day 28. Interestingly, in regional lymph nodes, the number of CD8^+^ cells was found to be significantly increased by the treatment with calcitriol (from 21–33 day of experiment) (Figure S7F). Moreover, in regional lymph nodes, the level of *Vdr* and immunosuppressive *Spp1* was found to be increased significantly after treatment with calcitriol and its analogs. In the tumor tissue, we also observed changed expressions of cytokines, considered to be involved in antitumor immune response; IFN-γ enhanced secretion, but decreased IL-1β expression after treatment with calcitriol and its analogs. However, the IL-10, involved in the tumor-induced immunosuppression, was found to be increased after treatment with calcitriol and its analogs. These results, taken together with the results from our previous study, which showed an increased level of OPN and decreased level of TGF-β in tumor tissue after treatment [22], revealed a lot of contradictory processes induced by calcitriol and its analogs, giving the negative final result in our experimental model.

In the lung tissue, in correlation with increased *Spp1* and *Tgfb* levels (in this study), we detected an increased lung metastatic foci formation [22]. Therefore, upregulated level of both *Spp1* and *Tgfb* by calcitriol and its analogs treatment in lung tissue, as well as *Spp1* in lymph nodes, are responsible for metastatic immunosuppressive niche formation and facilitation of metastasis. However, the cellular source of an increased OPN and TGF-β level should be analyzed in further studies. We demonstrated that granulocytes (neutrophils), which are the primary cells infiltrating lung tissue in 4T1 tumor-bearing mice [41], were found to be in the same number in the lung of 4T1 tumor-bearing mice after treatment with calcitriol and its analogs as in control mice. However, we observed a diminished percentage of I-CAM (CD54^+^) and CXCR4 (CD184^+^) expressing neutrophils in blood (Figure S6), which suggests their diminished antitumor potential [76]. Although the 4T1 cells were not sensitive to the treatment with calcitriol and its analogs in vitro [22], we cannot exclude these cells as the tumor-derived source of OPN and TGF-β in in vivo studies after indirect stimulation by other cells/cytokines in the body. A detailed characteristic of lung infiltrating cells is necessary to indicate the cells responsible for the increased level of pro-metastatic cytokines in this tissue.

## 4. Materials and Methods

### 4.1. Plasma, Tissues, and Cells

We harvested the cells, plasma, and tissues from 4T1 tumor-bearing mice with orthotopically transplanted mammary gland cancer cells (1 × 10^4^ cells per mice) [21]. Because of the ethical reasons, we decided to collect all of the biological material from the same mice and perform analysis without repeating animal experiments. All methods used were performed according to European Union (EU) Directive 2010/63/EU on the protection of animals used for scientific purposes and was approved by the First Local Committee for Experiments with the Use of Laboratory Animals, Wroclaw, Poland (No. of permission: 40/2014, 16/07/2014).

Calcitriol (1,25(OH)2D3) and its analogs, namely PRI-2191 and PRI-2205, were obtained from the Pharmaceutical Research Institute, Warsaw, Poland. We used six- to eight-week-old female BALB/c mice weighing 20–25 g (obtained from the Center of Experimental Medicine of the Medical University of Bialystok, Poland). Mice were subcutaneously (s.c.) treated with 80% propylene glycol (vehicle control), calcitriol (0.5 µg/kg), PRI-2191 (1.0 µg/kg), and PRI-2205 (10.0 µg/kg) thrice a week from the day 7 after transplantation of tumor cells. On days 0, 7, 14, 21, 28, and 33 after transplantation, blood was harvested under anesthesia prior to performing euthanasia of the mice [21]. Subsequently, the tumor, spleen, regional lymph nodes (axillary and inguinal), lungs, and liver were harvested for further analyses.

### 4.2. Flow Cytometry

#### Blood Cells, Splenocytes, and Lymph Nodes

To obtain the suspension of mononuclear cells and granulocytes, whole blood samples were centrifuged at 400× *g* for 30 min at RT using a gradient density Ficoll Paque Premium (GE Healthcare, Chicago, IL, USA) and the samples were subsequently frozen. The single-cell suspension of spleen and lymph nodes was prepared by passing through sterile nylon filters on a petri dish and then centrifuged twice at 192× *g* for 7 min at 4 °C. The cells (1 × 10^6^) suspended in phosphate buffered saline (PBS) with 2% fetal bovine serum (FBS; GE Healthcare, Chicago, IL, USA) were incubated with CD16/CD32 antibody to block the Fc receptors. After incubation, the splenocytes, mononuclear blood cells, and lymph nodes were stained with the anti-mouse conjugated antibodies as follows: rat CD8a-BV510, rat CD4-PE-Cy7, rat CD19-PE, hamster CD3e-APC, rat CD25-BV421, and rat CD335(NKp46)-FITC (BD Biosciences, Franklin Lakes, NJ, USA). Granulocytes were stained with rat CD45-PerCP-Cy5.5, rat Ly6G-Ly6C-APC, rat CD184-PE, and hamster CD54-FITC (BD Biosciences, Franklin Lakes, NJ, USA). Prior to analysis, the cells were washed with PBS containing 2% FBS (centrifuged at 192× *g* for 7 min at 4 °C). For data analysis, a BD LSR Fortessa cytometer with FACSDiva V8.0.1 software (BD Biosciences, Franklin Lakes, NJ, USA) was used.

### 4.3. Splenocytes Culture

Splenocytes (2 × 10^6^ cells/mL) were stimulated with lipopolysaccharide (LPS) from *Escherichia coli* (0.5 µg/mL) and concanavalin A (ConA) from *Canavalia ensiformis* (1 µg/mL) (both from Sigma-Aldrich, Saint Louis, MO, USA) for 48 h. Next, the supernatants were collected for further analyses.

### 4.4. Cytokine Array

Supernatants obtained from stimulated splenocytes culture were analyzed with proteome profiler arrays (Proteome Profiler Mouse Cytokine Array Kit, Panel A; R&D Systems, Minneapolis, MN, USA) according to the enclosed instructions. Pixel densities on X-ray film were collected using a multifunctional scanning device Samsung SLC460 (Samsung, Suwon, Korea) or Image Station 4000 MM PRO (Carestream Health, Rochester, NY, USA), after which image analysis was performed (ImageJ 1.48v). For each spot, the final level of optical density was determined as a factor acquired by subtracting the background optical level and dividing by values obtained from the non-treated mice (named as day 0).

### 4.5. Real-Time PCR Array

TRIzol (TRI Reagent; Sigma-Aldrich, Saint Louis, MO, USA) was used for the total RNA extraction according to the manufacturer’s recommendations. Quantity and purity of RNA were determined spectrophotometrically at 260 nm using NanoDrop 2000 (Thermo Fisher Scientific, Waltham, MA, USA). The quality of RNA was verified optionally by agarose gel electrophoresis. Expression of mRNA was quantified by real-time PCR through a ViiA™ 7 Real-Time PCR System (Thermo Fisher Scientific, Waltham, MA, USA) with SYBR green chemistry (Qiagen, Hilden, Germany).

#### 4.5.1. Lymph Nodes and Splenocytes

Synthesis of first-strand cDNA has been done using the compatible RT First Strand Kit (Qiagen, Hilden, Germany), eventually achieving 0.5 µg of cDNA (six mice pooled per group) for a single reaction. Mouse T Helper Cell Differentiation RT^2^ Profiler Array was acquired from Qiagen Company (Hilden, Germany). A list of genes is shown in Table S8. The conditions for PCR amplification were as follows: 95 °C for 10 min (1 cycle), 95 °C for 15 s (40 cycles), and 60 °C for 1 min. Relative quantification (RQ) values of the target genes were defined using ΔΔCt values with reference to beta-2 microglobulin (*B2m*); glyceraldehyde-3-phosphate dehydrogenase (*Gapdh*); and heat shock protein 90 alpha, class B member 1 (*Hsp90ab1*) genes for lymph nodes, and to actin beta (*Actb*) and *B2m* for splenocytes. Data were analyzed using Qiagen online software suitable for the purchased kit (Qiagen, Hilden, Germany).

#### 4.5.2. Lung Tissue

RNA and cDNA from lung tissue for real-time PCR arrays were prepared according to the method described in our previous study [22]. Data were analyzed using DataAssist 3.01 software (freeware by Applied Biosystems, Foster, CA, USA) with reference to ribosomal protein L13A (*Rpl13a*). The list of genes is shown in Table S9.

### 4.6. Real-Time PCR on Lung, Lymph Nodes, and Splenocytes

The isolation of RNA and synthesis of cDNA were performed as described previously [21]. Real-time PCR reaction was performed using specific TaqMan primers coding following genes: *Spp1* (Mm00436767_m1) and *Tgfb1* (Mm01178820_m1) for lung cDNA, and *Spp1* (Mm00436767_m1) and *Vdr* (Mm00437297_m1) for cDNA of spleen and lymph nodes (Thermo Fisher Scientific). Briefly, 25 or 40 ng of cDNA (lung or lymph nodes/spleen specimens, respectively) was used for a single reaction. Each sample was performed in triplicates. Data were analyzed using the comparative ΔΔCt method by DataAssist 3.01 software in comparison to endogenous controls: ribosomal protein L13A (*Rpl13a*, Mm01612987_g1) for lung, and beta-2 microglobulin (*B2m*, Mm00437762_m1) for lymph nodes and spleen samples.

### 4.7. ELISA

Quantitative determination of IFN-γ, TGF-β, IL-1β, IL-2, IL-4, IL-5, IL-6, IL-10, IL-17A, KC, OPN, and G-CSF cytokines using ELISA was performed according to the kit manufacturer’s instructions (R&D Systems, Minneapolis, MN, USA; eBioscience, Vienna, Austria, respectively). The expression of proteins was analyzed in plasma, tumor tissue, lung homogenates, and the spleen culture supernatants. Tumor and lung tissue specimens were stored at −80 °C. Preparation of tissue samples for ELISA and the method of protein determination have been described previously [22].

### 4.8. Histopathological Examination of Lung Tissue: Granulocytes Count

To evaluate infiltration of granulocytes into the lungs, tissue sections were isolated on day 28 and fixed in 4% buffered formalin. Lung tissue specimens were paraffin embedded, cut into 4-μm slices, and stained with hematoxylin and eosin. Granulocyte accumulation was examined based on cell morphology and scored according to the following scheme: 0—none, 1—slight, 2—average, and 3—intense, by an experienced pathologist. Microphotographs were subjected to a computer-assisted image analysis as previously described [77]. BX53 optical microscope (Olympus, Tokyo, Japan) with the CellA software (Olympus Soft Imaging Solution GmbH, Münster, Germany) was used.

### 4.9. Statistical Evaluation

STATISTICA version 10 (StatSoft Inc., Palo Alto, CA, USA) or GraphPad Prism 7.01 (GraphPad Software Inc., La Jolla, CA, USA) were used for statistical analysis. The assumptions of analysis of variance (ANOVA) were tested via Shapiro–Wilk’s normality test and Bartlett’s test. In the figure legends, specific tests used for data analysis are indicated. *p* value < 0.05 was considered as significant.

## 5. Conclusions

Thus, we conclude that the treatment of 4T1 tumor-bearing mice with calcitriol and its analogs drive the establishment of tumor-conducive milieu through the impact on immune system. This phenomenon is manifested by increased Th2 and Treg signature in lymph nodes and spleen, which may be supported by the action of calcitriol and its analogs toward granulocytes (decreasing the percentage of activated I-CAM^+^ and CXCR4^+^ granulocytes) in the blood. Furthermore, a decrease in NK CD335^+^ cells in the spleen and lymph nodes may contribute to the stimulated metastatic potential of 4T1 mammary gland tumor cells growing in mice treated with calcitriol and its analogs. Moreover, an increase in the expression of *Spp1* and *Tgfb* in the lung triggered by the applied treatment is responsible for the immunosuppressive metastatic niche formation. Thus, the results of this study indicate that in the case of breast cancer, which shows severe inflammatory response in addition to resistance to treatment with vitamin D compounds, further studies are necessary to determine the efficacy of vitamin D compounds in the treatment of this type of cancer.

## Supplementary Materials

Supplementary materials can be found at http://www.mdpi.com/1422-0067/19/7/2116/s1.

## Abbreviations

ACTB	actin, beta
ANGPT1	angiopoietin 1
ASB2	ankyrin repeat and SOCS box containing 2
B2M	beta-2 microglobulin
BCG	bacillus Calmette-Guerin
C/EBP	CCAAT/enhancer binding protein
CACNA1F	calcium voltage-gated channel subunit alpha1 F
CCL11	chemokine (C-C motif) ligand 7
CCL7	chemokine (C-C motif) ligand 7
CCR3	chemokine (C-C motif) receptor 3
ConA	Concanavalin A
CSF2	colony stimulating factor 2 (granulocyte-macrophage)
CST7	cystatin F (leukocystatin)
CTGF	connective tissue growth factor
CXCL-1	chemokine (C-X-C motif) ligand 1
CYP27B1	cytochrome P450 family 27 subfamily B member 1
FLT1	vascular endothelial growth factor receptor 1
FOS1	FOS like 1
FOXP3	forkhead box P3
GAPDH	glyceraldehyde-3-phosphate dehydrogenase
GATA4	GATA binding protein 4
G-CSF	granulocyte-colony stimulating factor
GM-CSF	granulocyte-macrophage colonystimulating factor
GPI	glucose phosphate isomerase
HOXA3, HOXA10	homeobox A3, A10
HSP90AB1	heat shock protein 90 alpha, class B member 1
IFN-γ	interferon gamma
IL	interleukin
IL-12p70	interleukin 12 subunit p70
Il12rb2	interleukin 12 receptor, beta 2
Il17re	interleukin 17 receptor E
Il1r1	interleukin 1 receptor type 1
Irf4, Irf8	interferon regulatory factor 4 and 8
LPS	lipopolysaccharide
MCP-5	monocyte chemotactic protein 5
M-CSF	macrophage colony-stimulating factor
METAP2	methionyl aminopeptidase 2
MIG	macrophage-induced gene
MIP	macrophage inflammatory protein
NF2	neurofibromin 2
NK	natural killer cells
Nr4a1, Nr4a3	nuclear receptor subfamily 4 group A members 1 and 3
OPN	osteopontin
p27KIP1	cyclin dependent kinase inhibitor
PSGFA	platelet-derived growth factor α
PTGRD2	prostaglandin D2 receptor 2
PTH	parathormone
REL	reticuloendotheliosis oncogene, NF-κB subunit
RORA	RAR-related orphan receptor alpha
RORC	RAR-related orphan receptor gamma
SDF-1	stromal cell-derived factor 1
sICAM-1	soluble intercellular adhesion molecule-1
Spp1	osteopontin encoded gene
TBX21	T-box 21
TGF-β	transforming growth factor β
TGIF1	TGFB-induced factor homeobox 1
TH	T helper lymphocytes
TIMP-1	tissue inhibitor of metalloproteinases
TNF	tumor necrosis factor
Tregs	regulatory T cells
TREM-1	triggering receptor expressed on myeloid cells
UTS2	urotensin 2
VDR	vitamin D receptor
VEGFA	vascular endothelial growth factor A
ZEB1	zinc finger E-box binding homeobox 1
