# Supplementary material for: Calcitriol and Its Analogs Establish the Immunosuppressive Microenvironment That Drives Metastasis in 4T1 Mouse Mammary Gland Cancer
Source: Int J Mol Sci. 2018 Jul 20;19(7):2116. doi: 10.3390/ijms19072116 (PMC6073894; doi:10.3390/ijms19072116)
Supplement: Supplementary file 1 [file ijms-19-02116-s001.pdf]

Calcitriol and its analogs establish the immunosuppressive microenvironment that drives metastasis in 4T1 mouse mammary gland cancer

Agata Pawlik[1], Artur Anisiewicz[1], Beata Filip-Psurska[1], Marcin Nowak[2], Eliza Turlej[1], Justyna Trynda[1], Joanna Banach[1], Paweł Gretkierewicz[1], Joanna Wietrzyk[1*]

[1]Department of Experimental Oncology, Hirszfeld Institute of Immunology and Experimental Therapy, Polish Academy of Sciences, Wroclaw, Poland

[2]Faculty of Veterinary Medicine, Wroclaw University of Environmental and Life Sciences, Wroclaw, Poland

*wietrzyk@iitd.pan.wroc.pl

1 Supplementary Materials and Methods

1.1 Blood morphology

Complete blood count analysis was evaluated in each blood sample using the hematology analyzer Mythic 18 (C2 Diagnostics, Montpellier, France).

2 Supplementary Results

2.1 Phenotype of peripheral blood granulocytes

The population of Ly6G-6C positive cells with high SCC parameter (Ly6G-6C^{+}SCC^{high}) was significantly higher in mice treated with calcitriol and with PRI-2205. Similar tendency was observed in mice treated with PRI-2191 (Figure S1A). Among the Ly6G-6C^{+}SCC^{high} cell population, all compounds, but only PRI-2205 in a significant manner, decreased the percentage of CD54^{+} cells (Figure S1B). PRI-2205 also significantly decreased the percentage of CD184^{+} cells within Ly6G-6C^{+}SCC^{high} population (Figure S1C).

3 Supplementary Figures and Tables

3.1 Supplementary Figures

Supplementary Figure S1. Calcitriol, PRI-2191, and PRI-2205 increased lymphocyte and monocyte percentage with a parallel decrease in granulocytes percentage in mice bearing 4T1 mammary gland tumors.

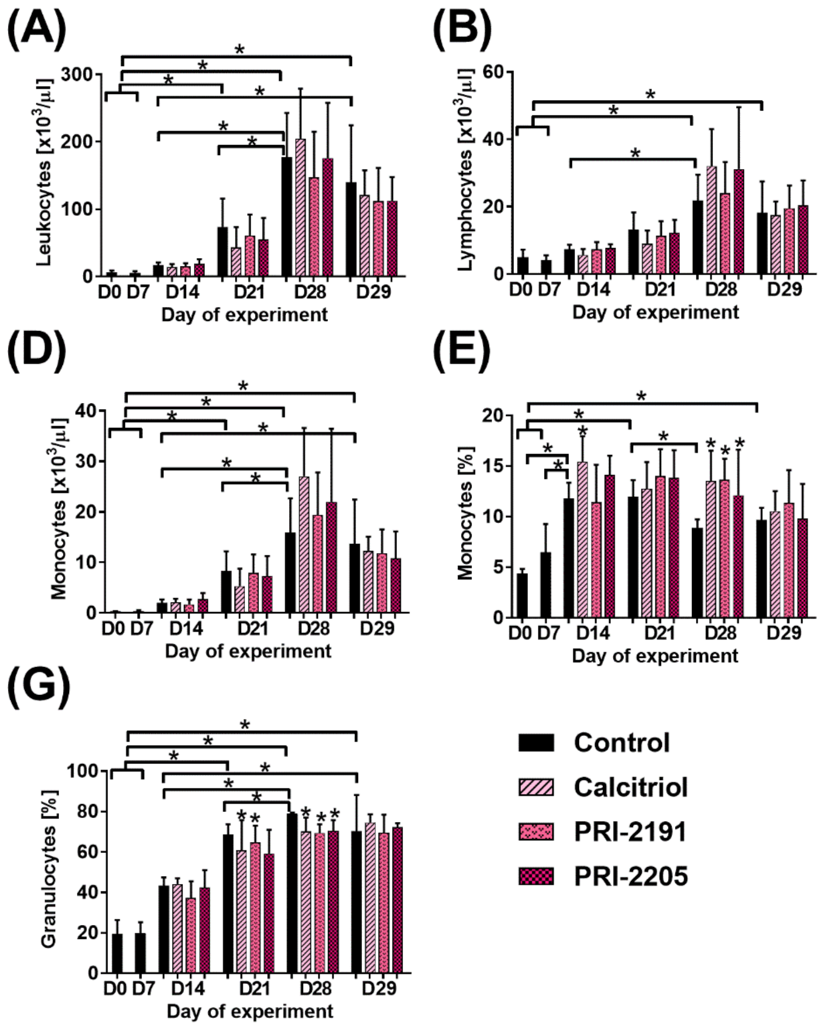

(A) General white blood cell count (WBC); the number (B) and the percentage (C) of: lymphocytes, (D) and (E) monocytes, (F) and (G) granulocytes. From day 7, vitamin D analogs were administered subcutaneously (s.c.) thrice a week. The single dose of compounds was as follows: calcitriol, 0.5 µg/kg; PRI-2191, 1.0 µg/kg; and PRI-2205, 10.0 µg/kg. Number of mice was 9–12 per group. The blood morphology was evaluated in each blood sample using the Mythic 18 automatic analyzer. Data are presented as mean ± SD. Statistical analysis: Kruskal–Wallis multiple comparison test. *p<0.05.

Supplementary Figure S2. Selected blood morphological parameters of mice bearing 4T1 mammary gland tumors and treated with calcitriol and its analogs: PRI-2191 and PRI-2205.

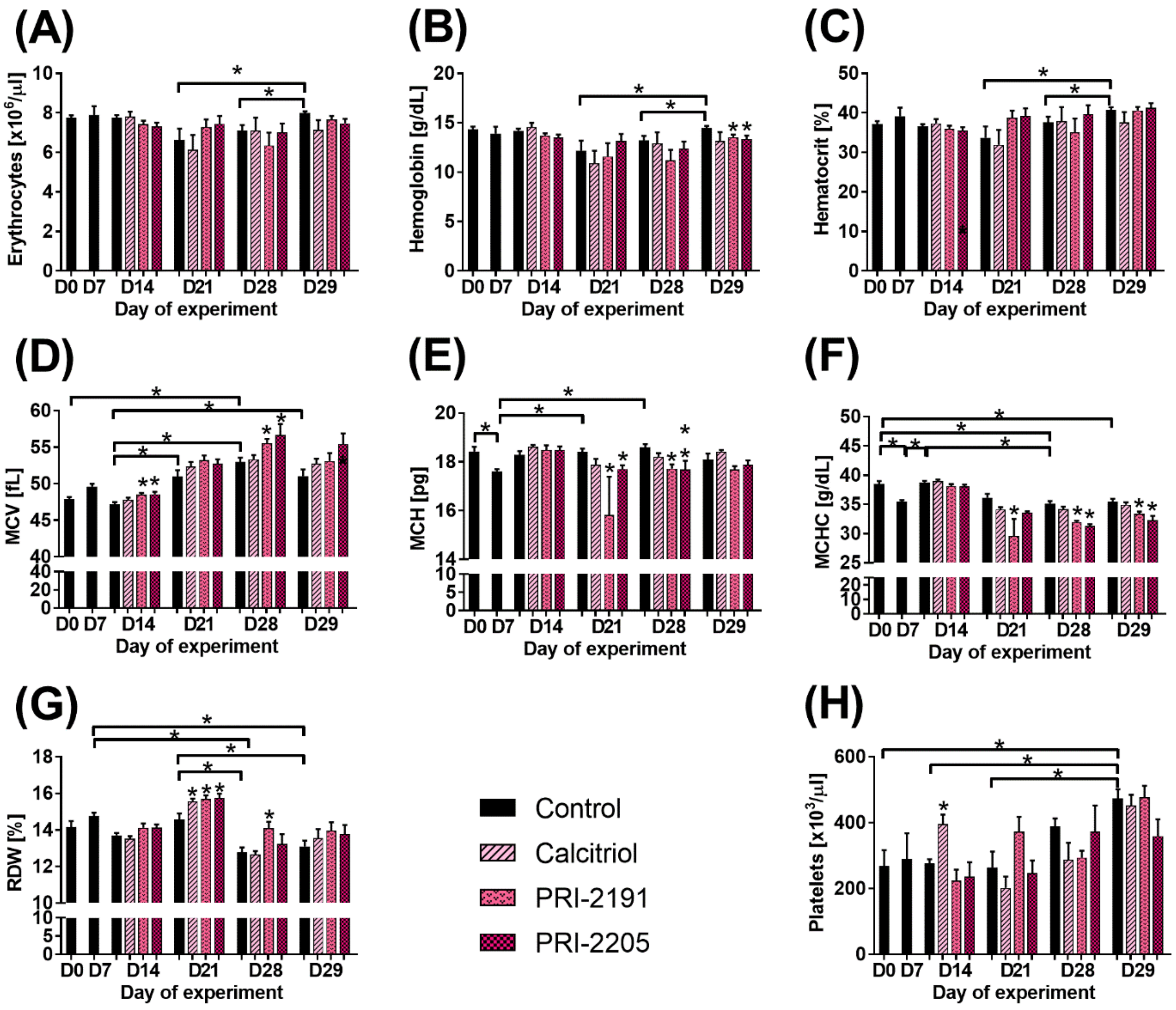

(A) Erythrocytes number; (B) hemoglobin (Hb); (C) hematocrit; (D) mean cell volume (MCV); (E) mean corpuscular hemoglobin (MCH); (F) mean corpuscular hemoglobin concentration (MCHC); (G) red distribution width (RDW); (H) platelets number. Mice were inoculated orthotopically with 4T1 cells on day 0. From day 7 (7 days after tumor inoculation), vitamin D analogs were administered subcutaneously (s.c.) thrice a week. The single dose of compounds were as follows: calcitriol, 0.5 μg/kg; PRI-2191, 1.0 μg/kg; and PRI-2205, 10.0 μg/kg. Number of mice were 6 per group. Data are presented as mean with standard deviation. Statistical analysis: Kruskal–Wallis multiple comparison test. *$P<0.05$ as compare to control mice on appropriate day or as indicated.

Supplementary Figure S3. Selected cytokine levels in plasma and supernatants from lipopolisaccharide (LPS)- or Concanavalin A (ConA)-stimulated splenocytes.

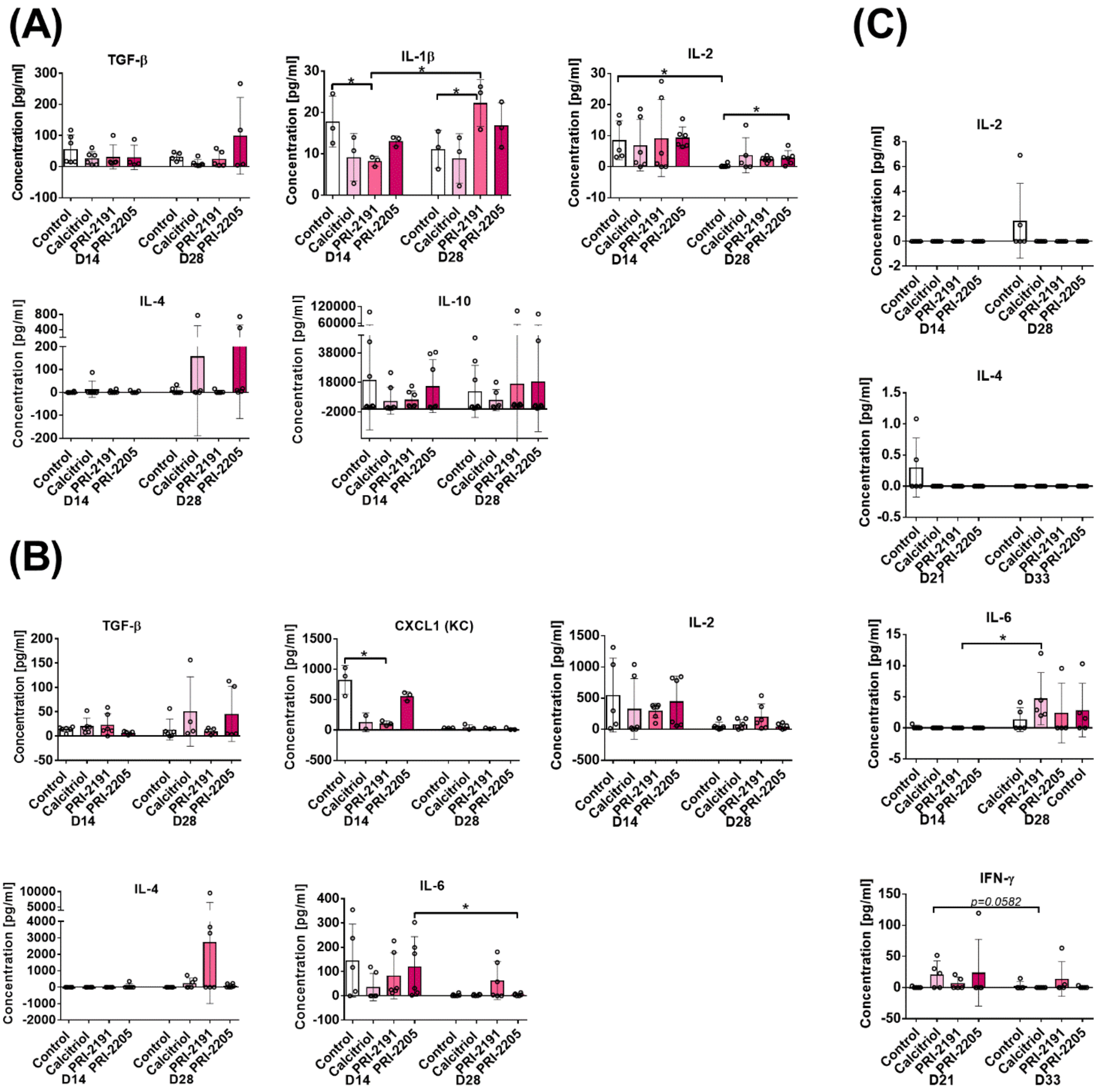

Supernatants obtained from spleen cells (harvested on days 14 and 28) stimulated with LPS (A) and ConA (B). Plasma from mice harvested on days 14 or 21 and 28 or 33 (C). Samples were analyzed with ELISA tests. Number of samples analyzed was 2–6 per group. Data are presented as mean ± SD and individual sample results. Statistical analysis: Kruskal–Wallis multiple comparison test. *p<0.05.

Supplementary Figure S4. Expression of selected cytokines in tumor tissue.

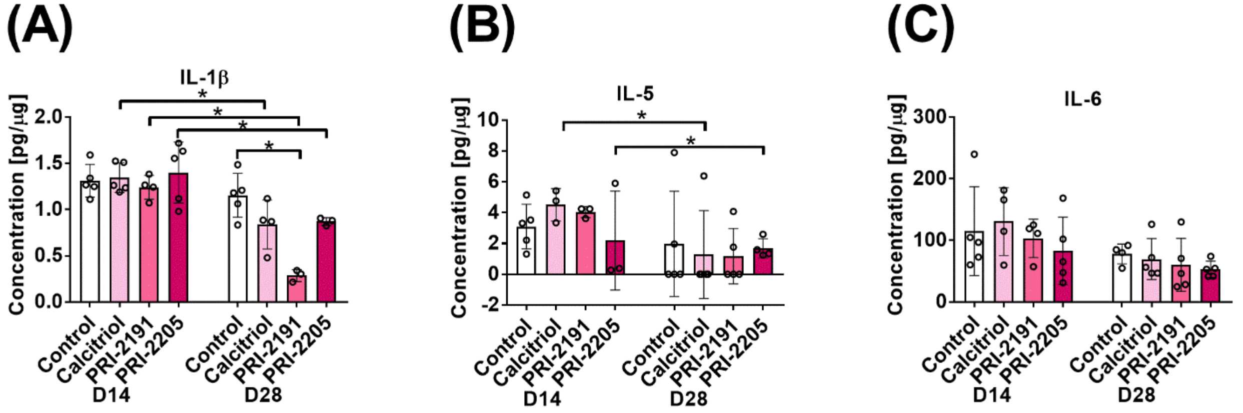

(A) IL-1β. (B) IL-5. (C) IL-6. Tumors harvested on day 14 and 28 were homogenized and lysed. Supernatants were analyzed with ELISA tests. Number of samples analyzed was 3–5 per group. Data are presented as mean ± SD and individual sample results. Statistical analysis: Kruskal–Wallis multiple comparison test. *$p<0.05$.

Supplementary Figure S5. Increase in the mRNA of acute phase proteins in the liver of mice bearing 4T1 mammary gland tumors treated with calcitriol, PRI-2191, and PRI-2205.

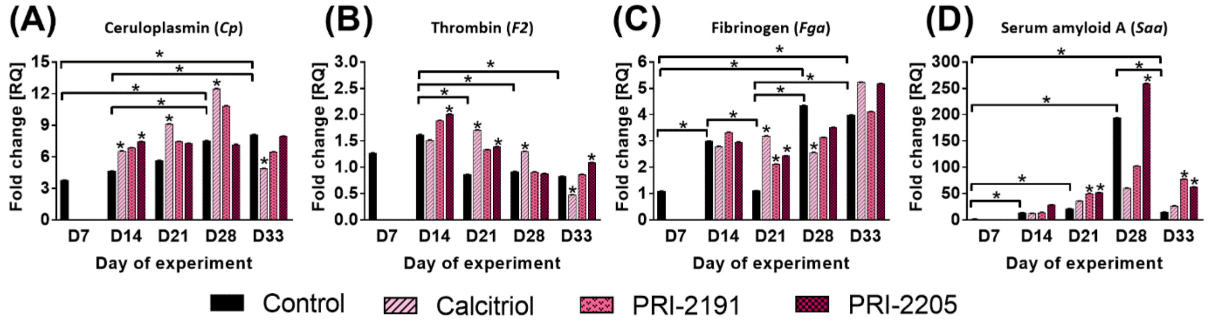

Real-time PCR analysis of four selected genes. (A) Ceruloplasmin (*Cp*), (B) Coagulation factor II, thrombin (*F2*), (C) Fibrinogen alpha chain (*Fga*), and (D) Serum amyloid A (*Saa*). Real-time PCR reaction was performed using specific primers coding following genes: *Cp* (Mm01289313_m1), *F2* (Mm00438843_m1), *Fga* (Mm00802584_m1), and *Saa* (Mm04208126_m1). Briefly, 50 ng of cDNA was used for a single reaction and each sample was performed in triplicate in a single experiment. Data were analyzed using comparative ΔΔCt method by DataAssist 3.01 software in comparison to endogenous control: hypoxanthine phosphoribosyltransferase 1 (*Hprt1*, Mm00446968_m1). Mice were orthotopically inoculated with 4T1 cells on day 0. From day 7 (7 days after tumor inoculation), vitamin D analogs were administered subcutaneously (s.c.) thrice a week. A single dose of compounds were as follows: calcitriol, 0.5 µg/kg; PRI-2191, 1.0 µg/kg; and PRI-2205, 10.0 µg/kg. Number of mice: 9–12 per group. Data are presented as mean ± SD. Statistical analysis: Kruskal–Wallis multiple comparison test. *$p<0.05$.

Supplementary Figure S6. Peripheral blood granulocytes phenotype in mice bearing 4T1 mammary gland tumors treated with calcitriol, PRI-2191, and PRI-2205 (day 28).

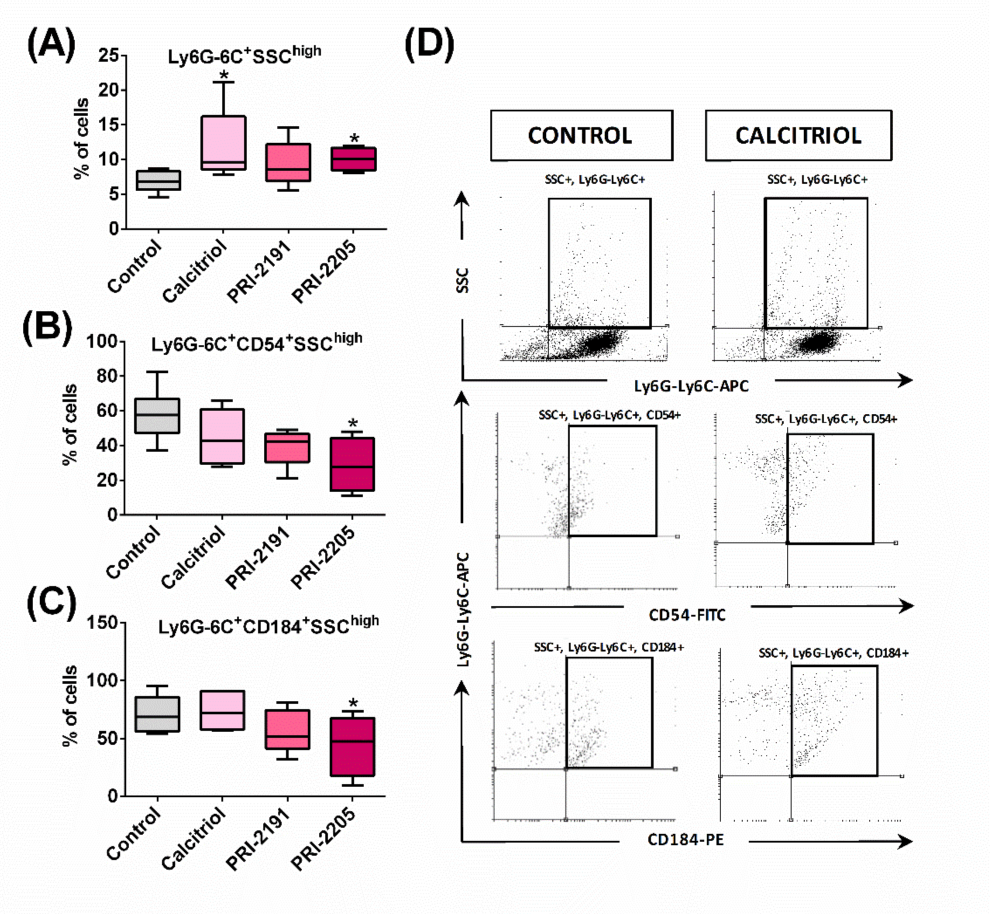

(A) Ly6G-6C+SCChigh mature granulocytes. (B) Ly6G-6C+CD54+SCChigh (C) Ly6G-6C+CD184+SCChigh. (D) Representative dot plots of selected analysis performed on day 28. Data for calcitriol as example are shown. Number of samples analyzed was 4–6 per group. CD54+ and CD184+ granulocytes were gated in SSChigh Ly6G-Ly6C+ population. Data were analyzed using the FACS Diva software. Data are presented as median line with min to max whiskers and 25–75% percentiles. Statistical analysis: Kruskal–Wallis multiple comparison test. *p<0.05.

Supplementary Figure S7. Phenotype of regional lymph nodes lymphocytes in mice bearing 4T1 mammary gland tumors treated with calcitriol, PRI-2191, and PRI-2205.

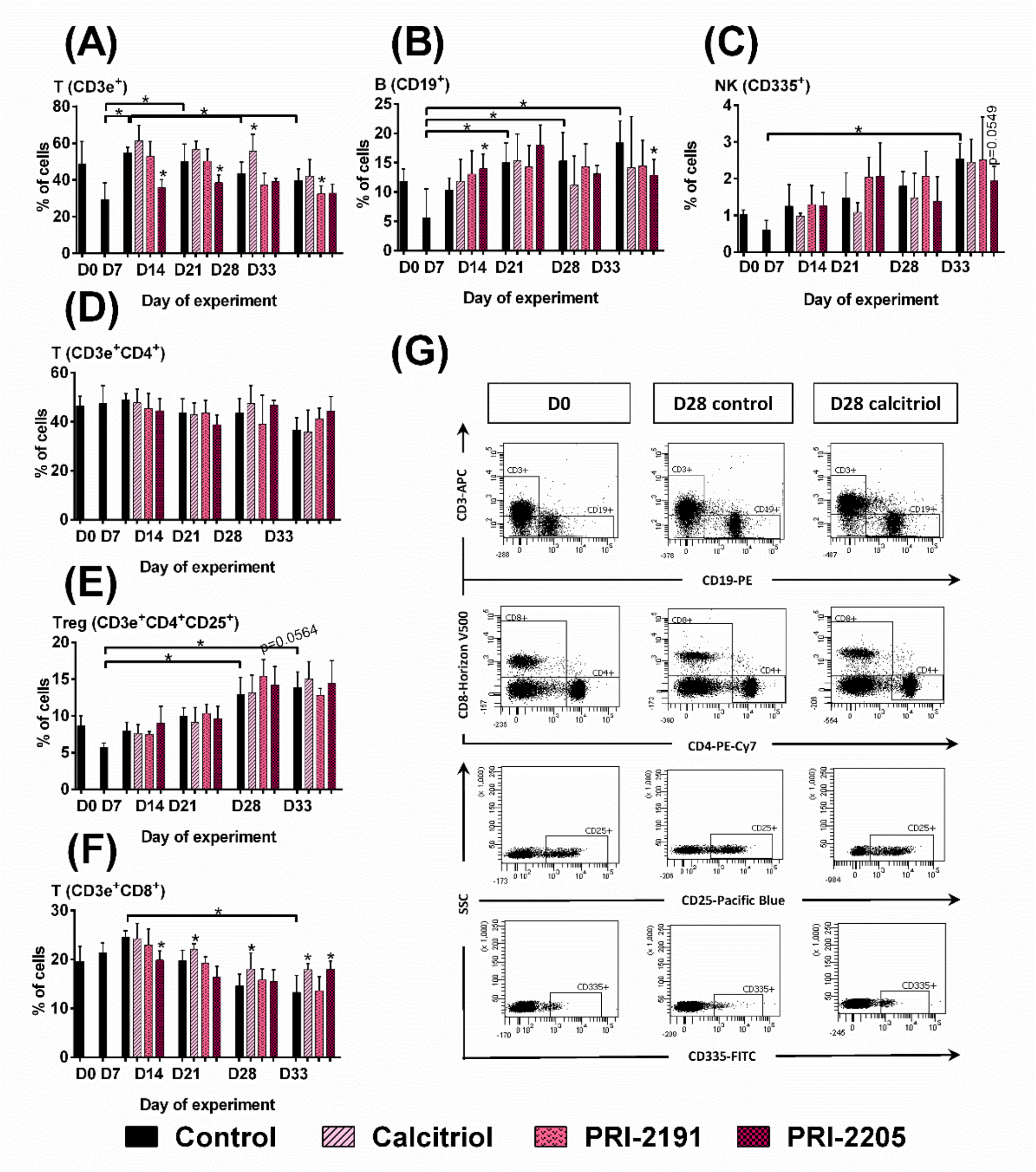

(A) T lymphocytes CD3e+. (B) B lymphocytes CD19+. (C) NK cells CD335+. (D) TCD4+ lymphocytes. (E) TCD4+CD25+ lymphocytes. (F) TCD8+ lymphocytes. (G) Representative dot plots of selected analysis performed on day 28. Six samples per group was analyzed, except: D0 = 2 and D7 = 3. Data analysis was performed using Becton Dickinson FACS Fortessa cytometer with FACSDiva software. Shown data represent mean ± SD. Statistical analysis: Kruskal–Wallis multiple comparison test. *$p<0.05$.

3.2 Supplementary Tables

Table S1. The fold change values of genes associated with precursor T cells differentiation in splenocytes samples from 4T1 tumor bearing mice treated with calcitriol or its analogs.

Gene	Calcitriol		PRI-2191		PRI-2205	
	D21	D28	D21	D28	D21	D28
Asb2	2.403	0.399	2.244	0.736	1.734	0.172
Cacna1f	1.127	1.006	1.389	0.645	0.805	0.357
Ccl11	1.877	3.821	37.502	0.891	8.350	2.973
Ccl5	1.835	0.586	0.950	1.790	1.092	0.623
Ccl7	20.283	0.421	718.457	0.848	22.735	1.586
Ccr3	2.667	0.654	1.097	0.687	2.940	0.378
Ccr4	1.275	0.564	7.324	1.174	0.613	1.355
Ccr6	1.426	0.325	3.600	1.630	0.442	0.921
Cebpb	2.531	1.580	0.083	0.575	1.444	0.102
Chd7	1.914	1.520	4.638	0.509	0.721	0.153
Csf2	4.144	1.546	26.864	4.072	20.050	1.746
Fasl	1.314	0.900	12.786	1.173	1.175	1.293
Fosl1	2.358	1.023	1.893	0.706	1.974	0.866
Foxp3	2.958	1.096	8.829	0.896	1.304	0.681
Gata3	2.038	0.817	1.455	0.418	0.972	0.289
Gata4	0.718	0.784	37.502	1.226	1.249	3.015
Gfi1	0.547	1.851	0.322	0.357	0.305	1.817
Ptgdr2	4.452	0.554	26.461	0.647	3.382	0.404
Havcr2	2.714	1.288	4.150	0.948	1.377	1.249
Hopx	1.271	1.495	6.179	0.737	1.921	2.957
Hoxa10	0.377	6.067	5.354	1.882	0.144	2.556
Hoxa3	0.646	3.653	11.007	1.226	0.367	10.585
Icos	1.277	0.352	1.031	0.962	0.186	0.071
Id2	1.246	1.681	0.785	0.746	0.750	1.604
Ifng	2.876	0.981	2.366	1.601	2.248	0.927
Igsf6	0.861	1.243	0.958	0.808	0.994	1.458
Ikzf2	2.388	0.673	2.746	1.049	0.941	0.506
Il12b	6.414	0.081	37.502	2.584	8.184	0.312
Il12rb2	3.963	0.554	20.624	0.280	0.772	0.404
Il13	0.718	2.517	37.502	3.844	1.249	3.724
Il13ra1	1.651	0.966	1.598	0.709	1.314	1.015
Il17a	0.718	0.784	37.502	1.226	1.249	3.015
Il17re	1.424	1.964	8.803	1.013	1.677	8.485
Il18	1.652	0.495	9.805	0.887	3.525	1.815
Il18r1	0.932	1.442	1.428	1.055	0.723	2.551
Il18rap	0.735	1.677	0.544	0.666	1.330	1.463
Il1r1	4.356	0.628	12.066	0.787	2.024	0.909
Il1r2	0.984	2.934	1.211	0.844	1.068	3.136
Il1rl1	2.134	1.239	2.738	2.270	2.565	1.205

Il2	3.488	0.668	37.502	1.066	1.249	2.491
Il21	1.197	1.081	22.145	0.676	0.738	3.969
Il2ra	1.412	0.854	1.371	0.516	0.883	0.707
Il4	2.591	1.779	14.039	0.948	0.974	10.196
Il4ra	1.599	1.105	0.066	0.706	1.422	0.149
Il5	1.538	0.784	37.502	1.226	2.231	3.224
Il9	0.718	0.784	37.502	1.226	1.249	7.205
Irf1	1.680	0.618	2.446	0.918	1.355	0.381
Irf4	3.636	0.328	0.858	0.813	1.361	0.175
Irf8	2.265	0.851	0.151	0.631	1.015	0.150
Jak1	1.054	1.050	7.362	1.304	0.940	2.033
Lrrc32	0.961	1.156	32.662	3.991	1.603	3.706
Maf	1.845	0.639	0.484	0.577	1.778	0.912
Myb	0.613	1.400	0.368	0.624	0.633	3.182
Nfatc1	1.584	0.951	1.178	1.036	1.293	0.865
Nfatc2	2.119	0.745	3.025	1.024	1.202	0.617
Nfatc2ip	1.126	1.020	0.825	0.632	0.879	0.426
Nr4a1	11.904	0.539	4.153	0.552	8.755	0.054
Nr4a3	2.393	0.233	5.710	0.741	1.403	0.254
Perp	0.718	0.608	37.502	0.637	1.249	2.969
Pkd2	1.326	1.135	1.518	0.666	1.407	0.957
Pou2f2	2.685	0.697	1.369	0.814	1.637	0.289
Pparg	1.098	0.690	4.236	0.829	2.466	2.349
Rel	3.438	0.552	15.543	0.718	2.359	0.622
Rora	1.590	1.200	8.765	1.033	1.235	1.639
Rorc	2.178	0.999	15.387	0.860	1.544	2.010
Runx1	2.072	0.943	0.854	0.611	1.284	0.587
Runx3	1.937	0.968	0.316	0.651	0.539	0.786
Socs1	1.676	0.959	0.459	0.719	0.838	0.631
Socs5	1.366	0.972	0.543	0.824	0.940	0.471
Stat1	1.024	0.732	1.574	0.859	0.842	0.797
Stat4	0.917	1.076	0.833	1.299	0.988	2.120
Stat6	1.368	1.178	2.144	0.623	0.632	0.120
Tbx21	1.789	0.513	5.301	0.359	0.564	0.728
Tgif1	4.189	0.517	2.687	0.760	1.785	0.155
Tlr4	1.242	0.927	0.929	0.723	1.010	0.728
Tlr6	1.240	1.042	0.200	0.917	1.826	1.030
Tmed1	1.356	1.356	1.478	0.600	1.625	0.312
Tnf	6.963	0.467	0.645	1.208	4.159	0.125
Tnfrsf9	1.631	0.941	1.920	4.069	0.418	0.372
Tnfsf11	1.613	1.164	2.218	1.813	1.590	2.940
Trp53inp1	1.830	1.346	0.325	0.819	1.592	1.039
Uts2	1.508	2.004	37.502	1.950	1.559	3.015
Zbtb7b	0.885	1.207	0.207	0.465	0.644	0.586
Zeb1	0.718	0.784	37.502	1.226	1.249	3.015

Spleen specimens were collected on the days 21 and 28 (after inoculation with 4T1 cells) from mice treated with calcitriol or its analogs and control group receiving vehicle. Real-time PCR screening was performed using

Mouse T Helper Cell Differentiation RT² Profiler Array (Qiagen, Hilden, Germany) including 84 key genes and 5 housekeeping genes in the set. Data shows a mean relative quantification (RQ) values. Fold-change (RQ) of target genes was defined using double delta Ct method in reference to actin, beta (Actb) and beta-2 microglobulin (B2m) for splenocytes samples. Then the results were adjusted to the values obtained for the control group within the day 21 or 28th of the experiment for each treatment group. Data analysis was acquired using Qiagen online software suitable for purchased kit (Qiagen, Hilden, Germany). PCR amplification cycles were as follows 95 °C for 10 s and 58 °C for 45 s (50 cycles). We used 0,5 μg of cDNA (6 mice pooled per group) for a single reaction.

Table S2. Pixel densities of plasma cytokines acquired with *Mouse Cytokine Array Panel A* (A-E).

A.

Cytokine	D0		D7		
	Mean	SD	Mean	SD	Factor
BLC	6.519	1.503	10.188	1.918	1.563
C5/C5a	130.931	0.442	144.681	9.953	1.105
G-CSF	2.500	0.752	4.906	0.000	1.963
GM-CSF	2.794	0.124	1.482	1.061	0.530
I-309	2.894	0.301	1.069	1.150	0.369
Eotaxin	1.507	0.018	0.631	0.035	0.419
sICAM-1	101.213	2.289	93.100	2.678	0.920
IFN-γ	6.469	2.227	2.781	1.203	0.430
IL-1α	6.681	0.017	2.700	0.503	0.404
IL-1β	2.425	0.380	1.169	1.025	0.482
IL-1ra	9.488	1.759	2.331	2.157	0.246
IL-2	9.050	1.440	2.337	1.370	0.258
IL-3	2.737	0.680	3.669	1.326	1.341
IL-4	7.112	0.062	7.431	0.707	1.045
IL-5	2.356	0.089	2.831	0.743	1.202
IL-6	2.012	0.397	0.512	0.751	0.255
IL-7	2.838	0.045	0.525	0.556	0.185
IL-10	1.981	0.318	-0.088	0.981	-0.044
IL-13	3.494	0.336	2.062	1.546	0.590
IL-12p70	3.000	1.211	0.950	1.051	0.317
IL-16	5.769	0.442	2.543	0.371	0.441
IL-17	3.406	0.035	0.343	1.909	0.101
IL-23	4.050	1.388	1.881	1.114	0.465
IL-27	4.525	0.238	2.194	0.865	0.485
IP-20	3.144	0.760	3.031	0.211	0.964
I-TAC	4.225	0.628	2.569	1.645	0.608
KC	2.544	1.095	1.850	0.079	0.727
M-CSF	10.162	1.246	8.731	0.583	0.859
JE	21.588	0.309	6.194	1.803	0.287
MCP-5	3.356	1.573	0.606	0.265	0.181
MIG	2.163	0.115	0.912	0.521	0.422
MIP-1α	2.425	1.565	1.688	0.786	0.696
MIP-1β	3.469	0.248	0.531	0.601	0.153
MIP-2	3.125	0.416	0.350	0.893	0.112
RANTES	15.918	0.177	0.337	0.981	0.021
SDF-1	22.700	0.893	4.537	0.327	0.200
TARC	1.456	0.371	2.663	0.397	1.829
TIMP-1	4.356	0.000	4.232	0.354	0.971
TNF-α	1.844	0.407	0.625	0.840	0.339
TREM-1	1.813	0.981	1.237	0.080	0.682
REF	178.071	11.274	182.323	6.490	1.024

B.

Cytokine	CONTROL					
	D21			D33		
	Mean	SD	Factor	Mean	SD	Factor
**BLC**	35.313	5.020	5.417	40.250	4.110	6.175
**C5/C5a**	108.751	1.909	0.831	101.131	0.547	0.772
**G-CSF**	108.775	2.599	43.519	105.250	0.875	42.108
**GM-CSF**	8.225	4.119	2.944	4.119	2.563	1.474
**I-309**	4.588	0.584	1.585	1.925	0.327	0.665
**Eotaxin**	2.925	1.167	1.942	-0.100	0.574	-0.066
**sICAM-1**	109.181	8.742	1.079	85.200	0.911	0.842
**IFN-γ**	7.819	6.302	1.209	3.156	2.652	0.488
**IL-1α**	15.951	0.530	2.387	8.619	0.124	1.290
**IL-1β**	5.788	0.124	2.387	19.056	0.124	7.860
**IL-1ra**	52.294	0.981	5.512	59.031	0.549	6.222
**IL-2**	11.200	0.105	1.238	1.288	1.087	0.142
**IL-3**	7.812	1.008	2.854	3.956	1.202	1.445
**IL-4**	15.944	3.014	2.242	5.394	0.071	0.758
**IL-5**	8.319	0.698	3.531	4.013	0.274	1.703
**IL-6**	4.632	1.140	2.302	2.175	0.521	1.081
**IL-7**	4.363	0.601	1.537	3.112	0.008	1.097
**IL-10**	1.807	0.327	0.912	-0.526	0.363	-0.265
**IL-13**	13.182	4.800	3.773	1.481	0.247	0.424
**IL-12p70**	1.656	1.247	0.552	0.169	0.477	0.056
**IL-16**	18.750	1.026	3.250	22.281	0.158	3.863
**IL-17**	3.856	0.787	1.132	2.912	0.716	0.855
**IL-23**	6.126	1.061	1.513	0.719	0.530	0.178
**IL-27**	5.257	0.557	1.162	3.931	1.025	0.869
**IP-20**	7.800	0.600	2.481	6.713	1.264	2.135
**I-TAC**	10.857	0.734	2.570	12.994	2.280	3.075
**KC**	55.475	2.245	21.810	20.006	0.106	7.866
**M-CSF**	76.438	4.613	7.522	55.081	6.736	5.420
**JE**	35.644	2.890	1.651	30.819	0.018	1.428
**MCP-5**	5.787	1.856	1.724	3.200	1.440	0.953
**MIG**	4.375	0.336	2.023	1.675	0.133	0.775
**MIP-1α**	2.200	0.105	0.907	0.519	0.442	0.214
**MIP-1β**	1.369	0.238	0.395	1.594	0.194	0.459
**MIP-2**	2.375	0.777	0.760	1.594	0.336	0.510
**RANTES**	8.700	2.263	0.547	4.388	0.009	0.276
**SDF-1**	75.319	2.166	3.318	35.150	2.184	1.548
**TARC**	8.663	1.114	5.952	8.357	0.937	5.741
**TIMP-1**	57.838	8.256	13.278	66.481	3.871	15.262
**TNF-α**	17.838	0.264	9.676	13.462	0.239	7.302
**TREM-1**	20.363	1.838	11.234	26.013	4.110	14.352
**REF**	189.756	10.363	1.066	143.412	25.643	0.805

C.

Cytokine	CALCITRIOL					
	D21			D33		
	Mean	SD	Factor	Mean	SD	Factor
**BLC**	10.719	1.034	1.644	43.576	6.249	6.685
**C5/C5a**	79.194	14.098	0.605	107.594	24.130	0.822
**G-CSF**	81.063	3.147	32.431	68.725	2.130	27.495
**GM-CSF**	2.988	1.131	1.070	4.332	0.070	1.551
**I-309**	2.357	0.292	0.814	3.738	0.168	1.292
**Eotaxin**	1.719	0.044	1.141	2.532	0.689	1.680
**sICAM-1**	107.363	5.551	1.061	89.538	1.564	0.885
**IFN-γ**	6.125	5.568	0.947	3.788	1.264	0.586
**IL-1α**	9.044	0.981	1.354	6.763	0.257	1.012
**IL-1β**	3.156	0.150	1.302	12.663	0.151	5.223
**IL-1ra**	38.875	2.086	4.097	53.569	1.237	5.646
**IL-2**	2.675	0.972	0.296	3.307	0.689	0.365
**IL-3**	2.181	0.256	0.797	3.657	0.354	1.336
**IL-4**	5.656	0.911	0.795	5.026	0.928	0.707
**IL-5**	2.369	0.009	1.005	4.463	0.027	1.894
**IL-6**	0.937	0.390	0.466	5.482	0.230	2.724
**IL-7**	2.756	0.150	0.971	4.813	0.044	1.696
**IL-10**	1.082	0.026	0.546	2.213	0.009	1.117
**IL-13**	3.076	0.548	0.880	3.007	0.088	0.861
**IL-12p70**	1.137	0.742	0.379	1.888	0.397	0.629
**IL-16**	7.100	0.407	1.231	10.332	0.141	1.791
**IL-17**	1.256	0.168	0.369	3.363	0.221	0.987
**IL-23**	1.856	0.044	0.458	2.763	0.080	0.682
**IL-27**	4.500	0.460	0.995	3.938	0.080	0.870
**IP-20**	2.751	0.619	0.875	4.226	0.450	1.344
**I-TAC**	5.475	0.106	1.296	9.144	0.707	2.164
**KC**	18.132	3.120	7.129	11.688	0.593	4.595
**M-CSF**	39.081	0.946	3.846	25.513	3.014	2.511
**JE**	23.038	0.760	1.067	20.938	0.008	0.970
**MCP-5**	3.306	0.911	0.985	4.444	0.778	1.324
**MIG**	2.076	0.725	0.960	2.488	0.079	1.151
**MIP-1α**	1.082	0.521	0.446	1.882	0.371	0.776
**MIP-1β**	0.588	0.070	0.169	1.325	0.098	0.382
**MIP-2**	0.762	0.212	0.244	1.901	0.186	0.608
**RANTES**	2.257	0.238	0.142	5.757	0.159	0.362
**SDF-1**	22.882	2.131	1.008	25.169	0.832	1.109
**TARC**	1.375	0.177	0.945	6.126	0.221	4.209
**TIMP-1**	21.675	1.344	4.976	45.151	5.259	10.365
**TNF-α**	4.950	0.742	2.685	3.650	0.256	1.980
**TREM-1**	8.513	0.230	4.697	10.494	0.653	5.790
**REF**	169.198	11.558	0.950	166.946	13.607	0.938

D.

Cytokine	PRI-2191					
	D21			D33		
	Mean	SD	Factor	Mean	SD	Factor
**BLC**	12.937	1.493	1.985	23.650	1.291	3.628
**C5/C5a**	73.719	9.476	0.563	102.413	4.490	0.782
**G-CSF**	53.406	4.154	21.366	89.900	1.079	35.967
**GM-CSF**	3.156	1.184	1.130	3.169	0.787	1.134
**I-309**	2.413	0.221	0.834	1.350	0.247	0.467
**Eotaxin**	1.375	0.592	0.912	0.244	0.168	0.162
**sICAM-1**	99.937	0.822	0.987	88.450	1.256	0.874
**IFN-γ**	3.769	1.785	0.583	1.813	1.131	0.280
**IL-1α**	6.594	0.247	0.987	6.532	0.221	0.978
**IL-1β**	4.256	0.106	1.755	14.625	1.450	6.032
**IL-1ra**	22.037	0.133	2.323	31.644	1.493	3.335
**IL-2**	3.313	0.151	0.366	0.281	0.185	0.031
**IL-3**	4.400	1.512	1.608	1.600	0.177	0.585
**IL-4**	18.463	1.777	2.596	2.350	0.017	0.330
**IL-5**	6.212	0.751	2.637	1.806	0.027	0.767
**IL-6**	1.363	0.026	0.677	1.488	0.036	0.739
**IL-7**	2.250	0.026	0.793	2.038	0.318	0.718
**IL-10**	0.869	0.035	0.438	0.332	0.363	0.167
**IL-13**	6.044	1.290	1.730	1.613	0.124	0.462
**IL-12p70**	1.106	0.160	0.369	-0.075	0.460	-0.025
**IL-16**	8.431	0.194	1.461	13.088	0.265	2.269
**IL-17**	2.431	0.636	0.714	1.331	0.027	0.391
**IL-23**	2.219	0.301	0.548	0.581	0.327	0.143
**IL-27**	2.350	0.310	0.519	1.206	0.911	0.267
**IP-20**	3.800	0.080	1.209	3.081	0.593	0.980
**I-TAC**	6.488	0.380	1.536	8.025	0.443	1.899
**KC**	22.694	0.159	8.922	9.019	0.910	3.546
**M-CSF**	38.163	0.769	3.755	29.988	0.407	2.951
**JE**	22.307	1.715	1.033	19.007	2.696	0.880
**MCP-5**	2.737	1.069	0.816	2.681	0.557	0.799
**MIG**	2.838	0.026	1.312	2.194	0.522	1.015
**MIP-1α**	1.800	0.150	0.742	0.556	0.027	0.229
**MIP-1β**	0.993	0.070	0.286	0.175	0.106	0.050
**MIP-2**	1.381	0.530	0.442	0.713	0.425	0.228
**RANTES**	3.994	0.070	0.251	3.825	0.071	0.240
**SDF-1**	29.294	1.644	1.290	16.688	1.785	0.735
**TARC**	2.869	0.071	1.971	3.144	0.115	2.160
**TIMP-1**	24.681	1.131	5.666	22.813	0.672	5.237
**TNF-α**	9.038	0.186	4.902	5.588	0.035	3.031
**TREM-1**	11.644	0.442	6.424	19.450	0.354	10.731
**REF**	172.954	16.214	0.971	150.189	40.145	0.843

E.

Cytokine	PRI-2205					
	D21			D33		
	Mean	SD	Factor	Mean	SD	Factor
**BLC**	19.525	2.077	2.995	56.432	1.979	8.657
**C5/C5a**	107.007	2.988	0.817	100.313	15.513	0.766
**G-CSF**	98.944	1.167	39.586	99.307	1.132	39.731
**GM-CSF**	5.126	1.247	1.835	4.138	1.141	1.481
**I-309**	4.219	0.035	1.458	4.207	0.141	1.454
**Eotaxin**	2.713	0.380	1.801	1.844	0.018	1.224
**sICAM-1**	122.626	13.621	1.212	120.957	1.432	1.195
**IFN-γ**	4.950	2.820	0.765	2.600	1.140	0.402
**IL-1α**	9.138	1.511	1.368	8.776	0.415	1.314
**IL-1β**	4.256	0.071	1.755	18.695	1.432	7.711
**IL-1ra**	30.963	0.221	3.264	84.975	4.180	8.957
**IL-2**	3.813	0.539	0.421	1.875	0.522	0.207
**IL-3**	3.294	0.512	1.204	2.882	0.318	1.053
**IL-4**	5.695	0.088	0.801	3.463	0.291	0.487
**IL-5**	2.951	0.080	1.252	2.013	0.274	0.854
**IL-6**	1.419	0.372	0.705	1.119	0.300	0.556
**IL-7**	2.944	0.231	1.038	2.501	0.151	0.881
**IL-10**	1.094	0.159	0.552	0.726	0.097	0.366
**IL-13**	4.569	0.159	1.308	3.013	0.628	0.862
**IL-12p70**	0.919	0.460	0.306	0.663	0.256	0.221
**IL-16**	11.676	0.945	2.024	23.031	0.530	3.993
**IL-17**	2.088	0.027	0.613	2.007	0.053	0.589
**IL-23**	2.625	0.115	0.648	2.969	0.212	0.733
**IL-27**	2.000	0.115	0.442	1.376	0.238	0.304
**IP-20**	6.107	0.230	1.943	7.069	0.689	2.249
**I-TAC**	7.150	0.274	1.692	7.545	0.972	1.786
**KC**	24.538	1.989	9.647	13.875	0.663	5.455
**M-CSF**	59.413	0.716	5.847	43.625	0.327	4.293
**JE**	30.232	0.319	1.400	31.751	1.865	1.471
**MCP-5**	5.238	0.804	1.561	6.357	0.619	1.894
**MIG**	3.607	0.284	1.668	3.907	0.211	1.806
**MIP-1α**	1.444	0.141	0.596	1.244	0.194	0.513
**MIP-1β**	1.169	0.052	0.337	1.544	0.106	0.445
**MIP-2**	1.382	0.105	0.442	1.251	0.045	0.400
**RANTES**	4.563	0.574	0.287	7.232	1.149	0.454
**SDF-1**	45.550	2.413	2.007	37.688	0.787	1.660
**TARC**	2.894	0.371	1.988	3.988	0.699	2.740
**TIMP-1**	36.851	0.115	8.460	64.888	0.274	14.896
**TNF-α**	12.050	0.805	6.536	9.706	0.177	5.265
**TREM-1**	13.294	0.761	7.335	23.594	0.035	13.017
**REF**	183.781	10.745	1.032	171.892	16.768	0.965

Plasma specimens were collected on the days 0, 7, 21 and 33 (after inoculation with 4T1 cells) from mice treated with calcitriol or its analogs and control group receiving vehicle. Results were obtained using *Proteome Profiler*

Mouse Cytokine Array Kit, Panel A (R&D alp Systems. Inc. USA) according to the enclosed instruction. This array detects 40 mouse cytokines. chemokines. and acute phase proteins simultaneously. Pixel densities on developed X-ray film were collected using a multifunctional scanning device (Samsung SLC460) or Image Station 4000MM PRO (Carestream Health. Rochester. New York. USA) and image analysis software (ImageJ 1.48v). For each spot the final optical density level was determined as a factor acquired by subtracting the background optical level and dividing by values obtained from the untreated mice (Day 0).

Table S3. Pixel densities of cytokines contained in the supernatants from stimulated with lipopolisaccharide (LPS) splenocytes obtained using *Mouse Cytokine Array Panel A* (A-E).

A.

Cytokine	Day 0		Day 7		
	Mean	SD	Mean	SD	Factor
**BLC**	23.738	2.126	0.045	0.201	0.002
**C5/C5a**	5.514	0.433	-0.443	0.078	-0.080
**G-CSF**	81.026	4.795	5.761	0.837	0.071
**GM-CSF**	55.980	0.343	0.645	0.243	0.012
**I-309**	35.248	0.585	0.201	0.371	0.006
**Eotaxin**	1.832	0.564	-0.343	0.015	-0.187
**sICAM-1**	101.720	1.149	9.379	0.515	0.092
**IFN-γ**	34.038	3.560	0.550	0.239	0.016
**IL-1α**	70.394	2.505	2.563	0.518	0.036
**IL-1β**	65.182	0.888	3.630	0.016	0.056
**IL-1ra**	124.207	0.747	65.550	0.687	0.528
**IL-2**	54.187	6.538	1.617	0.378	0.030
**IL-3**	9.705	0.584	-0.473	0.049	-0.049
**IL-4**	8.972	0.560	-0.358	0.317	-0.040
**IL-5**	11.306	1.761	0.311	0.386	0.028
**IL-6**	74.049	0.567	0.548	0.170	0.007
**IL-7**	16.377	0.170	-0.075	0.155	-0.005
**IL-10**	72.831	4.371	2.771	0.279	0.038
**IL-13**	17.981	1.064	0.639	0.604	0.036
**IL-12p70**	32.327	4.586	1.731	0.280	0.054
**IL-16**	105.465	5.210	28.311	0.473	0.268
**IL-17**	43.160	5.823	1.840	0.059	0.043
**IL-23**	59.595	0.972	2.138	0.081	0.036
**IL-27**	18.794	2.734	0.859	1.161	0.046
**IP-20**	59.092	0.025	1.010	0.042	0.017
**I-TAC**	10.701	0.454	-0.554	0.077	-0.052
**KC**	131.671	5.156	71.225	1.881	0.541
**M-CSF**	55.022	16.395	0.251	0.541	0.005
**JE**	111.643	1.313	35.037	1.242	0.314
**MCP-5**	18.083	4.277	-0.259	0.091	-0.014

**MIG**	45.425	32.978	0.848	1.623	0.019
**MIP-1α**	136.452	0.658	120.183	13.653	0.881
**MIP-1β**	131.384	2.016	47.542	0.530	0.362
**MIP-2**	135.929	0.426	117.924	1.045	0.868
**RANTES**	133.400	0.702	66.125	0.808	0.496
**SDF-1**	86.462	7.575	10.235	2.345	0.118
**TARC**	3.405	1.497	-0.056	0.069	-0.016
**TIMP-1**	30.856	0.829	0.471	0.055	0.015
**TNF-α**	119.593	0.648	40.878	0.999	0.342
**TREM-1**	32.815	7.308	-0.044	0.295	-0.001
**REF**	135.998	0.349	130.278	1.557	0.958

B.

**Cytokine**	**CONTROL**					
	**Day 21**			**Day 33**		
	**Mean**	**SD**	**Factor**	**Mean**	**SD**	**Factor**
**BLC**	-0.897	0.004	-0.038	40.249	1.485	1.696
**C5/C5a**	-1.498	0.320	-0.272	16.813	0.697	3.049
**G-CSF**	62.953	5.863	0.777	129.958	5.928	1.604
**GM-CSF**	5.289	1.387	0.094	88.125	10.071	1.574
**I-309**	0.144	0.266	0.004	57.490	0.663	1.631
**Eotaxin**	-0.775	0.286	-0.423	12.282	0.857	6.706
**sICAM-1**	11.683	1.252	0.115	79.322	8.806	0.780
**IFN-γ**	0.418	0.230	0.012	37.058	4.029	1.089
**IL-1α**	41.006	2.080	0.583	96.893	4.443	1.376
**IL-1β**	77.673	1.078	1.192	126.021	2.314	1.933
**IL-1ra**	129.620	1.041	1.044	138.319	0.345	1.114
**IL-2**	39.297	43.091	0.725	63.048	12.612	1.164
**IL-3**	0.744	0.284	0.077	23.517	2.182	2.423
**IL-4**	-0.688	0.111	-0.077	24.120	0.416	2.688
**IL-5**	7.696	0.161	0.681	31.945	6.112	2.825
**IL-6**	43.243	0.483	0.584	119.355	5.639	1.612
**IL-7**	3.377	0.564	0.206	49.270	2.469	3.009
**IL-10**	58.105	0.875	0.798	112.557	4.528	1.545
**IL-13**	0.353	0.216	0.020	30.179	4.196	1.678
**IL-12p70**	9.321	0.943	0.288	25.600	4.332	0.792
**IL-16**	65.577	3.615	0.622	130.150	2.278	1.234
**IL-17**	13.103	2.432	0.304	85.816	7.847	1.988
**IL-23**	23.972	2.109	0.402	114.436	0.777	1.920
**IL-27**	3.715	3.411	0.198	34.448	11.399	1.833
**IP-20**	95.025	5.773	1.608	134.932	3.057	2.283
**I-TAC**	3.509	4.125	0.328	44.475	4.694	4.156
**KC**	114.070	9.272	0.866	136.731	1.455	1.038
**M-CSF**	16.740	12.671	0.304	82.967	13.839	1.508
**JE**	110.680	6.720	0.991	137.645	2.798	1.233
**MCP-5**	19.124	12.324	1.058	104.037	11.448	5.753
**MIG**	5.184	8.144	0.114	34.689	15.578	0.764

**MIP-1α**	124.862	2.483	0.915	138.259	0.735	1.013
**MIP-1β**	122.275	1.855	0.931	139.967	0.095	1.065
**MIP-2**	123.231	2.575	0.907	139.771	1.216	1.028
**RANTES**	118.067	2.157	0.885	138.355	0.370	1.037
**SDF-1**	33.734	22.956	0.390	80.278	12.758	0.928
**TARC**	3.262	1.727	0.958	30.755	4.536	9.032
**TIMP-1**	21.756	0.898	0.705	104.209	3.070	3.377
**TNF-α**	83.588	9.313	0.699	133.475	6.075	1.116
**TREM-1**	41.564	10.045	1.267	107.346	13.181	3.271
**REF**	117.227	6.539	0.862	139.261	0.602	1.024

C.

Cytokine	CALCITRIOL					
	Day 21			Day 33		
	Mean	SD	Factor	Mean	SD	Factor
**BLC**	14.644	0.342	0.617	11.067	0.516	0.466
**C5/C5a**	8.709	0.315	1.580	5.853	0.677	1.062
**G-CSF**	105.412	0.826	1.301	121.296	1.832	1.497
**GM-CSF**	50.630	0.303	0.904	60.817	1.724	1.086
**I-309**	31.048	1.619	0.881	26.361	0.919	0.748
**Eotaxin**	4.683	1.409	2.557	3.537	0.175	1.931
**sICAM-1**	75.841	2.232	0.746	63.663	0.775	0.626
**IFN-γ**	35.356	0.957	1.039	24.534	0.301	0.721
**IL-1α**	79.534	1.133	1.130	102.096	7.075	1.450
**IL-1β**	108.163	0.498	1.659	121.906	0.571	1.870
**IL-1ra**	137.018	1.547	1.103	137.518	0.344	1.107
**IL-2**	36.017	15.298	0.665	26.095	6.983	0.482
**IL-3**	11.482	0.645	1.183	5.579	0.651	0.575
**IL-4**	11.398	0.332	1.270	5.880	0.101	0.655
**IL-5**	25.857	0.433	2.287	18.462	10.945	1.633
**IL-6**	88.520	1.950	1.195	131.455	1.915	1.775
**IL-7**	34.407	0.012	2.101	37.198	5.612	2.271
**IL-10**	102.227	7.662	1.404	80.696	1.420	1.108
**IL-13**	23.740	6.194	1.320	34.898	3.450	1.941
**IL-12p70**	39.006	1.400	1.207	16.198	2.477	0.501
**IL-16**	126.816	3.970	1.202	126.957	0.655	1.204
**IL-17**	69.176	9.176	1.603	65.947	11.907	1.528
**IL-23**	94.952	10.462	1.593	72.408	5.806	1.215
**IL-27**	18.181	1.664	0.967	11.426	2.091	0.608
**IP-20**	126.821	1.500	2.146	109.271	3.434	1.849
**I-TAC**	20.682	1.537	1.933	6.561	0.979	0.613
**KC**	138.039	0.654	1.048	68.041	1.969	0.517
**M-CSF**	60.503	8.965	1.100	51.157	12.142	0.930
**JE**	138.504	0.083	1.241	140.285	0.744	1.257
**MCP-5**	62.477	12.436	3.455	73.380	18.259	4.058
**MIG**	43.675	32.420	0.961	32.230	19.721	0.710

**MIP-1α**	139.332	0.563	1.021	138.632	0.223	1.016
**MIP-1β**	140.568	0.396	1.070	139.671	0.295	1.063
**MIP-2**	140.664	0.011	1.035	95.301	8.422	0.701
**RANTES**	137.762	2.286	1.033	137.296	0.400	1.029
**SDF-1**	70.593	12.774	0.816	59.268	6.441	0.685
**TARC**	16.181	0.643	4.752	8.866	0.959	2.604
**TIMP-1**	59.710	0.934	1.935	52.598	0.454	1.705
**TNF-α**	125.770	4.511	1.052	126.441	7.226	1.057
**TREM-1**	74.956	9.144	2.284	70.558	10.769	2.150
**REF**	139.381	0.218	1.025	138.315	1.228	1.017

D.

**Cytokine**	**PRI-2191**					
	**Day 21**			**Day 33**		
	**Mean**	**SD**	**Factor**	**Mean**	**SD**	**Factor**
**BLC**	2.776	0.693	0.117	10.666	1.097	0.449
**C5/C5a**	1.772	0.091	0.321	4.183	0.855	0.759
**G-CSF**	60.192	4.340	0.743	94.889	2.193	1.171
**GM-CSF**	16.988	2.247	0.303	9.108	2.420	0.163
**I-309**	9.152	1.158	0.260	9.659	0.055	0.274
**Eotaxin**	2.819	0.303	1.539	4.288	0.528	2.341
**sICAM-1**	31.190	0.349	0.307	47.993	1.190	0.472
**IFN-γ**	5.538	0.376	0.163	5.235	0.519	0.154
**IL-1α**	29.717	0.426	0.422	45.406	3.761	0.645
**IL-1β**	72.399	6.403	1.111	100.828	2.266	1.547
**IL-1ra**	135.750	1.812	1.093	144.628	2.326	1.164
**IL-2**	7.784	4.562	0.144	8.032	4.586	0.148
**IL-3**	1.705	0.161	0.176	5.360	0.261	0.552
**IL-4**	2.318	0.163	0.258	6.401	0.732	0.713
**IL-5**	2.730	0.301	0.241	7.368	1.053	0.652
**IL-6**	26.862	2.949	0.363	38.931	0.110	0.526
**IL-7**	7.958	1.344	0.486	12.731	1.229	0.777
**IL-10**	76.837	4.441	1.055	97.113	5.599	1.333
**IL-13**	3.879	0.720	0.216	7.266	0.853	0.404
**IL-12p70**	16.630	3.757	0.514	4.636	0.057	0.143
**IL-16**	80.172	2.276	0.760	111.458	0.959	1.057
**IL-17**	19.959	1.897	0.462	19.127	2.979	0.443
**IL-23**	24.987	2.839	0.419	28.565	3.321	0.479
**IL-27**	4.325	1.663	0.230	4.296	1.293	0.229
**IP-20**	78.676	1.864	1.331	106.758	5.658	1.807
**I-TAC**	2.178	0.324	0.204	4.598	0.386	0.430
**KC**	86.618	15.143	0.658	123.545	1.126	0.938
**M-CSF**	19.014	2.169	0.346	11.705	3.752	0.213
**JE**	131.244	6.973	1.176	143.125	4.648	1.282
**MCP-5**	10.060	6.840	0.556	51.299	8.037	2.837
**MIG**	12.904	14.044	0.284	5.850	1.846	0.129
**MIP-1α**	143.602	0.934	1.052	119.366	9.129	0.875
**MIP-1β**	140.211	3.483	1.067	149.593	1.059	1.139

**MIP-2**	143.061	0.645	1.052	149.454	0.112	1.100
**RANTES**	138.197	1.986	1.036	142.195	3.965	1.066
**SDF-1**	46.739	8.979	0.541	48.983	11.902	0.567
**TARC**	2.312	0.542	0.679	6.136	1.146	1.802
**TIMP-1**	11.337	2.604	0.367	51.007	1.647	1.653
**TNF-α**	74.115	10.272	0.620	103.726	8.144	0.867
**TREM-1**	34.224	2.191	1.043	55.476	6.578	1.691
**REF**	143.687	0.636	1.057	143.200	0.716	1.053

E.

**Cytokine**	**PRI-2205**					
	**Day 21**			**Day 33**		
	**Mean**	**SD**	**Factor**	**Mean**	**SD**	**Factor**
**BLC**	3.286	0.084	0.138	3.750	0.354	0.158
**C5/C5a**	2.109	0.094	0.383	1.762	0.120	0.319
**G-CSF**	82.583	4.914	1.019	73.251	3.041	0.904
**GM-CSF**	16.169	0.068	0.289	19.561	0.137	0.349
**I-309**	2.662	0.058	0.076	2.639	0.115	0.075
**Eotaxin**	-0.648	0.353	-0.354	2.152	0.085	1.175
**sICAM-1**	37.650	1.421	0.370	39.604	3.356	0.389
**IFN-γ**	3.567	0.482	0.105	3.931	0.159	0.115
**IL-1α**	40.246	1.633	0.572	49.052	3.570	0.697
**IL-1β**	74.733	0.745	1.147	102.614	0.909	1.574
**IL-1ra**	121.997	5.846	0.982	141.772	1.662	1.141
**IL-2**	5.214	1.806	0.096	14.303	5.122	0.264
**IL-3**	2.932	0.396	0.302	3.733	0.024	0.385
**IL-4**	2.040	0.105	0.227	1.975	0.448	0.220
**IL-5**	12.436	1.683	1.100	2.775	0.087	0.245
**IL-6**	55.485	0.402	0.749	37.927	1.754	0.512
**IL-7**	8.533	1.777	0.521	9.596	1.621	0.586
**IL-10**	68.625	2.708	0.942	89.952	2.992	1.235
**IL-13**	3.270	0.898	0.182	4.574	1.725	0.254
**IL-12p70**	13.398	1.939	0.414	5.036	0.244	0.156
**IL-16**	89.897	1.066	0.852	103.306	4.699	0.980
**IL-17**	16.061	1.283	0.372	19.268	2.747	0.446
**IL-23**	30.755	1.270	0.516	44.801	1.926	0.752
**IL-27**	4.325	0.948	0.230	5.104	1.832	0.272
**IP-20**	78.240	0.540	1.324	120.390	3.420	2.037
**I-TAC**	2.494	0.424	0.233	2.442	0.405	0.228
**KC**	131.981	6.746	1.002	74.213	0.220	0.564
**M-CSF**	24.634	6.649	0.448	14.896	1.781	0.271
**JE**	134.656	0.076	1.206	134.001	3.113	1.200
**MCP-5**	8.113	6.913	0.449	66.237	16.965	3.663
**MIG**	19.404	22.149	0.427	5.916	5.411	0.130
**MIP-1α**	136.035	2.993	0.997	125.289	6.986	0.918
**MIP-1β**	137.810	0.503	1.049	141.594	0.791	1.078

**MIP-2**	136.390	1.242	1.003	134.307	1.081	0.988
**RANTES**	130.380	5.392	0.977	137.537	3.498	1.031
**SDF-1**	42.145	5.669	0.487	36.775	8.413	0.425
**TARC**	3.643	0.454	1.070	6.840	0.030	2.009
**TIMP-1**	9.209	0.192	0.298	37.433	0.808	1.213
**TNF-α**	109.950	3.451	0.919	98.079	13.105	0.820
**TREM-1**	49.217	5.267	1.500	56.408	3.613	1.719
**REF**	136.316	3.157	1.002	132.685	1.484	0.976

Spleen specimens were collected on the days 0, 7, 21 and 33 (after inoculation with 4T1 cells) from mice treated with calcitriol or its analogs and control group receiving vehicle and stimulated with lipopolisaccharide (LPS). Results were obtained using *Proteome Profiler Mouse Cytokine Array Kit. Panel A* (R&D alp Systems. Inc. USA) according to the enclosed instruction. This array detects 40 mouse cytokines. chemokines. and acute phase proteins simultaneously. Pixel densities on developed X-ray film were collected using a multifunctional scanning device (Samsung SLC460) or Image Station 4000MM PRO (Carestream Health. Rochester. New York. USA) and image analysis software (ImageJ 1.48v). For each spot the final optical density level was determined as a factor acquired by subtracting the background optical level and dividing by values obtained from the untreated mice (Day 0).

Table S4. Pixel densities of cytokines contained in the supernatants from stimulated with Concanavalin A (ConA) splenocytes obtained using *Mouse Cytokine Array Panel A*(A-E).

A.

Cytokine	Day 0		Day 7		
	Mean	SD	Mean	SD	Factor
**BLC**	24.093	0.862	2.192	0.351	0.091
**C5/C5a**	8.417	1.727	0.855	0.062	0.102
**G-CSF**	50.457	1.605	1.773	0.265	0.035
**GM-CSF**	97.510	5.016	14.988	0.163	0.154
**I-309**	28.431	0.304	0.901	0.525	0.032
**Eotaxin**	2.276	0.077	0.104	0.177	0.046
**sICAM-1**	92.882	6.220	7.951	0.736	0.086
**IFN-γ**	52.569	2.445	1.080	0.169	0.021
**IL-1α**	62.158	0.045	4.668	0.593	0.075
**IL-1β**	49.003	1.266	6.462	0.730	0.132
**IL-1ra**	114.766	8.124	74.239	3.758	0.647
**IL-2**	127.274	4.724	59.126	3.797	0.465
**IL-3**	111.816	5.187	23.161	1.334	0.207
**IL-4**	15.652	4.757	0.395	0.739	0.025
**IL-5**	6.058	0.721	0.775	0.733	0.128
**IL-6**	43.903	0.440	1.488	0.276	0.034
**IL-7**	14.703	3.291	3.180	1.543	0.216
**IL-10**	26.288	1.309	0.150	0.211	0.006
**IL-13**	42.653	3.433	0.379	0.518	0.009
**IL-12p70**	12.142	0.688	2.897	0.584	0.239
**IL-16**	99.521	4.315	32.383	0.738	0.325
**IL-17**	107.137	4.215	21.087	1.081	0.197
**IL-23**	46.775	6.078	4.856	0.489	0.104
**IL-27**	25.938	3.702	2.558	0.636	0.099
**IP-20**	94.847	2.882	19.585	1.882	0.206
**I-TAC**	12.013	1.787	0.269	0.076	0.022
**KC**	111.443	1.946	48.117	2.534	0.432
**M-CSF**	37.872	2.880	1.242	0.226	0.033
**JE**	97.958	1.675	49.514	0.665	0.505
**MCP-5**	8.066	1.554	0.744	0.473	0.092
**MIG**	24.013	7.953	1.336	1.095	0.056
**MIP-1α**	137.543	1.469	103.000	6.341	0.749
**MIP-1β**	101.336	5.985	47.186	1.578	0.466
**MIP-2**	131.549	3.461	87.477	1.523	0.665
**RANTES**	121.788	2.899	84.079	0.882	0.690
**SDF-1**	88.438	6.220	27.074	4.643	0.306
**TARC**	7.836	1.099	1.022	0.298	0.130
**TIMP-1**	24.295	0.123	0.791	0.386	0.033

**TNF-α**	108.639	2.019	32.804	1.766	0.302
**TREM-1**	23.288	4.076	1.256	0.048	0.054
**REF**	140.518	0.286	95.544	1.609	0.680

B.

Cytokine	CONTROL					
	Day 21			Day 33		
	**Mean**	**SD**	**Factor**	**Mean**	**SD**	**Factor**
**BLC**	1.964	0.124	0.082	9.132	0.640	0.379
**C5/C5a**	1.504	0.298	0.179	13.070	2.776	1.553
**G-CSF**	2.459	0.197	0.049	34.563	0.501	0.685
**GM-CSF**	3.760	0.549	0.039	33.974	1.755	0.348
**I-309**	2.062	0.645	0.073	40.241	0.247	1.415
**Eotaxin**	1.315	0.598	0.578	2.596	0.476	1.141
**sICAM-1**	2.678	0.098	0.029	73.587	3.337	0.792
**IFN-γ**	2.088	0.513	0.040	40.391	2.484	0.768
**IL-1α**	3.320	0.156	0.053	52.898	2.713	0.851
**IL-1β**	27.045	4.030	0.552	77.059	0.212	1.573
**IL-1ra**	116.177	3.326	1.012	131.521	3.677	1.146
**IL-2**	53.406	11.938	0.420	73.884	5.018	0.581
**IL-3**	19.171	1.611	0.171	12.788	5.773	0.114
**IL-4**	1.723	0.760	0.110	17.237	2.069	1.101
**IL-5**	0.876	0.020	0.145	1.412	0.675	0.233
**IL-6**	1.705	0.023	0.039	3.930	0.345	0.090
**IL-7**	2.017	0.055	0.137	28.324	0.960	1.926
**IL-10**	1.795	0.192	0.068	6.439	0.823	0.245
**IL-13**	1.308	0.497	0.031	15.238	0.235	0.357
**IL-12p70**	2.433	0.120	0.200	2.253	0.441	0.186
**IL-16**	28.853	2.651	0.290	122.546	8.124	1.231
**IL-17**	4.257	0.004	0.040	33.746	8.119	0.315
**IL-23**	10.800	1.223	0.231	66.402	0.091	1.420
**IL-27**	3.721	1.203	0.143	25.281	1.121	0.975
**IP-20**	87.991	0.165	0.928	73.708	0.723	0.777
**I-TAC**	3.394	3.031	0.282	14.531	2.326	1.210
**KC**	4.521	0.195	0.041	6.018	1.363	0.054
**M-CSF**	1.532	0.077	0.040	40.680	0.326	1.074
**JE**	76.260	2.775	0.778	134.581	0.633	1.374
**MCP-5**	9.063	4.183	1.124	37.650	25.427	4.668
**MIG**	1.305	0.681	0.054	16.966	0.221	0.707
**MIP-1α**	84.015	6.534	0.611	50.259	1.317	0.365
**MIP-1β**	87.521	0.823	0.864	44.651	0.512	0.441
**MIP-2**	82.005	1.203	0.623	28.013	0.211	0.213
**RANTES**	93.770	0.214	0.770	44.394	3.868	0.365
**SDF-1**	9.751	7.724	0.110	75.951	2.675	0.859
**TARC**	2.301	0.506	0.294	8.454	0.408	1.079
**TIMP-1**	1.189	0.992	0.049	41.542	2.132	1.710
**TNF-α**	6.130	0.508	0.056	58.223	1.808	0.536

**TREM-1**	2.662	0.046	0.114	78.023	1.206	3.350
**REF**	88.250	3.598	0.628	136.648	0.419	0.972

C.

**Cytokine**	**CALCITRIOL**					
	**Day 21**			**Day 33**		
	**Mean**	**SD**	**Factor**	**Mean**	**SD**	**Factor**
**BLC**	13.242	0.581	0.550	5.817	0.118	0.241
**C5/C5a**	10.658	2.647	1.266	1.799	0.083	0.214
**G-CSF**	11.465	0.349	0.227	5.917	0.489	0.117
**GM-CSF**	37.310	1.268	0.383	28.302	0.922	0.290
**I-309**	14.657	0.738	0.516	13.565	0.461	0.477
**Eotaxin**	1.897	0.210	0.833	0.049	0.074	0.022
**sICAM-1**	56.442	1.287	0.608	47.269	1.385	0.509
**IFN-γ**	27.064	0.423	0.515	24.701	1.400	0.470
**IL-1α**	36.810	0.101	0.592	37.452	1.046	0.603
**IL-1β**	58.780	0.642	1.200	59.075	3.366	1.206
**IL-1ra**	132.755	4.535	1.157	110.467	1.753	0.963
**IL-2**	90.919	8.637	0.714	70.303	2.147	0.552
**IL-3**	47.531	0.565	0.425	23.034	0.501	0.206
**IL-4**	9.493	2.686	0.607	2.411	0.479	0.154
**IL-5**	1.677	0.043	0.277	-0.301	0.013	-0.050
**IL-6**	4.554	0.491	0.104	3.810	0.375	0.087
**IL-7**	40.178	1.299	2.733	19.409	3.243	1.320
**IL-10**	10.304	0.421	0.392	3.323	1.002	0.126
**IL-13**	8.677	0.763	0.203	6.855	1.095	0.161
**IL-12p70**	2.599	0.228	0.214	0.846	0.211	0.070
**IL-16**	115.017	5.718	1.156	104.869	8.519	1.054
**IL-17**	33.365	1.054	0.311	29.288	5.408	0.273
**IL-23**	37.992	0.015	0.812	32.568	1.981	0.696
**IL-27**	13.614	1.388	0.525	7.515	0.559	0.290
**IP-20**	127.386	6.916	1.343	111.910	4.192	1.180
**I-TAC**	18.324	10.508	1.525	4.412	1.817	0.367
**KC**	22.240	0.293	0.200	1.918	0.146	0.017
**M-CSF**	33.146	0.282	0.875	13.195	3.215	0.348
**JE**	133.589	4.523	1.364	134.271	3.843	1.371
**MCP-5**	37.644	21.203	4.667	28.269	22.963	3.505
**MIG**	10.631	0.233	0.443	10.626	0.476	0.443
**MIP-1α**	61.942	0.710	0.450	32.127	0.808	0.234
**MIP-1β**	58.897	0.332	0.581	42.223	2.220	0.417
**MIP-2**	46.325	0.477	0.352	11.997	0.605	0.091
**RANTES**	85.271	1.199	0.700	48.638	1.066	0.399
**SDF-1**	60.584	4.829	0.685	59.313	0.689	0.671
**TARC**	9.118	0.339	1.164	7.971	0.105	1.017
**TIMP-1**	26.988	0.457	1.111	30.465	0.970	1.254
**TNF-α**	60.914	1.692	0.561	54.750	2.111	0.504

**TREM-1**	59.767	1.836	2.566	51.306	0.783	2.203
**REF**	135.487	1.611	0.964	137.360	1.259	0.978

D.

Cytokine	PRI-2191					
	Day 21			Day 33		
	Mean	SD	Factor	Mean	SD	Factor
**BLC**	2.382	0.446	0.099	2.220	0.082	0.092
**C5/C5a**	1.397	0.030	0.166	0.704	0.435	0.084
**G-CSF**	2.921	0.240	0.058	1.369	0.646	0.027
**GM-CSF**	13.741	0.917	0.141	3.130	0.062	0.032
**I-309**	2.824	0.249	0.099	3.283	0.199	0.115
**Eotaxin**	1.205	0.573	0.529	0.543	0.230	0.239
**sICAM-1**	21.056	0.353	0.227	24.149	0.798	0.260
**IFN-γ**	5.598	1.228	0.106	4.220	0.375	0.080
**IL-1α**	4.641	0.054	0.075	8.418	1.054	0.135
**IL-1β**	49.956	0.474	1.019	23.402	0.065	0.478
**IL-1ra**	126.837	7.783	1.105	121.442	6.689	1.058
**IL-2**	71.661	5.564	0.563	6.346	1.378	0.050
**IL-3**	43.191	0.405	0.386	2.682	0.710	0.024
**IL-4**	2.643	0.250	0.169	1.153	0.267	0.074
**IL-5**	0.221	0.110	0.036	-0.236	0.397	-0.039
**IL-6**	2.147	0.318	0.049	0.695	0.306	0.016
**IL-7**	24.555	3.367	1.670	3.358	0.101	0.228
**IL-10**	10.249	0.762	0.390	1.105	0.172	0.042
**IL-13**	3.331	0.152	0.078	1.877	0.021	0.044
**IL-12p70**	2.075	0.108	0.171	1.002	0.133	0.082
**IL-16**	78.181	0.857	0.786	95.354	6.852	0.958
**IL-17**	7.725	1.363	0.072	3.225	0.928	0.030
**IL-23**	17.526	2.184	0.375	10.943	2.028	0.234
**IL-27**	3.605	0.985	0.139	1.714	0.460	0.066
**IP-20**	128.356	1.276	1.353	28.399	0.038	0.299
**I-TAC**	3.305	2.157	0.275	1.534	0.143	0.128
**KC**	9.330	0.781	0.084	1.403	0.151	0.013
**M-CSF**	15.589	0.866	0.412	4.260	0.413	0.112
**JE**	138.875	1.716	1.418	88.865	4.107	0.907
**MCP-5**	11.493	6.698	1.425	2.180	1.597	0.270
**MIG**	2.702	0.726	0.113	1.910	0.414	0.080
**MIP-1α**	83.954	2.666	0.610	9.821	2.130	0.071
**MIP-1β**	73.258	1.255	0.723	7.792	1.104	0.077
**MIP-2**	51.434	0.275	0.391	3.847	0.584	0.029
**RANTES**	129.670	4.036	1.065	4.446	0.122	0.037
**SDF-1**	39.605	6.540	0.448	38.925	1.907	0.440
**TARC**	5.324	0.554	0.680	2.128	0.224	0.272
**TIMP-1**	5.970	0.411	0.246	4.093	0.076	0.168
**TNF-α**	52.882	0.093	0.487	7.961	0.772	0.073
**TREM-1**	34.659	0.778	1.488	37.152	0.299	1.595

**REF**	143.102	2.142	1.018	141.605	2.461	1.008

E.

**Cytokine**	**PRI-2205**					
	**Day 21**			**Day 33**		
	**Mean**	**SD**	**Factor**	**Mean**	**SD**	**Factor**
**BLC**	3.735	0.036	0.155	2.766	0.434	0.115
**C5/C5a**	3.060	0.379	0.364	1.321	0.469	0.157
**G-CSF**	3.050	0.272	0.060	0.417	0.098	0.008
**GM-CSF**	7.629	0.124	0.078	5.018	0.469	0.051
**I-309**	1.841	0.459	0.065	1.201	0.039	0.042
**Eotaxin**	0.982	0.129	0.431	-1.242	0.321	-0.545
**sICAM-1**	36.483	0.395	0.393	22.499	0.110	0.242
**IFN-γ**	3.627	0.257	0.069	1.634	0.577	0.031
**IL-1α**	10.298	1.227	0.166	2.724	0.721	0.044
**IL-1β**	40.951	0.467	0.836	33.414	2.347	0.682
**IL-1ra**	121.343	3.577	1.057	102.794	0.736	0.896
**IL-2**	26.605	3.942	0.209	42.834	2.633	0.337
**IL-3**	4.897	0.593	0.044	13.702	0.957	0.123
**IL-4**	2.651	0.216	0.169	0.835	0.482	0.053
**IL-5**	1.368	0.132	0.226	-0.303	0.032	-0.050
**IL-6**	1.256	0.108	0.029	-0.121	0.191	-0.003
**IL-7**	13.928	5.160	0.947	7.919	3.287	0.539
**IL-10**	1.886	0.108	0.072	0.359	0.036	0.014
**IL-13**	1.932	0.018	0.045	0.809	0.246	0.019
**IL-12p70**	1.606	0.039	0.132	0.248	0.177	0.020
**IL-16**	85.388	1.729	0.858	78.826	2.741	0.792
**IL-17**	3.931	0.271	0.037	6.251	0.552	0.058
**IL-23**	8.259	1.692	0.177	13.252	0.078	0.283
**IL-27**	4.757	1.199	0.183	2.497	0.541	0.096
**IP-20**	90.386	0.190	0.953	126.311	1.622	1.332
**I-TAC**	2.355	0.602	0.196	2.863	2.019	0.238
**KC**	5.457	0.110	0.049	0.520	0.108	0.005
**M-CSF**	6.662	0.015	0.176	3.992	0.865	0.105
**JE**	123.732	0.270	1.263	133.561	3.678	1.363
**MCP-5**	2.731	1.761	0.339	11.038	10.554	1.368
**MIG**	1.606	0.227	0.067	1.165	0.049	0.049
**MIP-1α**	23.294	1.662	0.169	12.280	0.373	0.089
**MIP-1β**	11.896	0.218	0.117	21.272	0.694	0.210
**MIP-2**	14.046	0.075	0.107	2.650	0.457	0.020
**RANTES**	48.356	1.208	0.397	50.628	0.052	0.416
**SDF-1**	44.500	2.239	0.503	21.742	0.425	0.246
**TARC**	3.736	0.339	0.477	6.003	1.320	0.766
**TIMP-1**	4.959	1.086	0.204	3.856	1.096	0.159
**TNF-α**	30.002	1.435	0.276	15.887	0.671	0.146
**TREM-1**	40.215	1.474	1.727	44.962	0.285	1.931

REF	134.553	0.662	0.958	134.718	1.594	0.959

Spleen specimens were collected on the days 0, 7, 21 and 33 (after inoculation with 4T1 cells) from mice treated with calcitriol or its analogs and control group receiving vehicle and stimulated with Concanavalin A (ConA). Results were obtained using *Proteome Profiler Mouse Cytokine Array Kit. Panel A* (R&D alp Systems. Inc. USA) according to the enclosed instruction. This array detects 40 mouse cytokines, chemokines, and acute phase proteins simultaneously. Pixel densities on developed X-ray film were collected using a multifunctional scanning device (Samsung SLC460) or Image Station 4000MM PRO (Carestream Health. Rochester. New York. USA) and image analysis software (ImageJ 1.48v). For each spot the final optical density level was determined as a factor acquired by subtracting the background optical level and dividing by values obtained from the untreated mice (Day 0).

Table S5. The fold change values of genes associated with precursor T cells differentiation in mouse lymph node samples from 4T1 tumor bearing mice treated with calcitriol or its analogs.

LYMPH NODES						
**Gene**	**Calcitriol**		**PRI-2191**		**PRI-2205**	
	**D14**	**D28**	**D14**	**D28**	**D14**	**D28**
Asb2	0.775	2.091	1.100	2.101	1.205	2.058
Cacna1f	2.292	1.937	1.057	1.933	4.895	2.025
Ccl11	0.749	1.002	0.612	0.931	0.727	0.731
Ccl5	1.371	1.141	1.676	1.165	1.636	0.782
Ccl7	2.497	1.784	3.229	1.109	3.220	1.666
Ccr3	0.718	0.417	0.707	0.527	0.969	0.491
Ccr4	0.726	0.806	0.706	0.860	0.698	0.585
Ccr6	0.914	0.607	0.798	0.792	1.036	0.738
Cebpb	1.025	3.788	0.927	2.456	0.883	4.005
Chd7	1.397	2.364	1.203	1.587	1.785	2.530
Csf2	0.417	2.909	0.389	1.214	6.436	4.031
Fasl	1.153	1.299	0.755	1.190	0.749	1.096
Fosl1	1.811	1.635	0.363	3.318	1.448	8.437
Foxp3	1.287	1.800	1.788	1.772	1.422	2.380
Gata3	0.951	1.128	1.487	1.211	1.590	1.234
Gata4	1.310	2.203	0.535	1.422	3.239	3.145
Gfi1	1.297	1.343	1.192	0.930	1.021	1.899
Ptgdr2	2.236	3.174	1.507	0.747	1.406	2.371
Havcr2	0.976	0.666	0.590	1.008	0.941	1.077
Hopx	0.663	1.243	0.776	1.010	1.093	1.236
Hoxa10	0.195	1.356	0.176	1.540	0.284	2.526
Hoxa3	0.604	2.118	0.342	0.685	0.893	1.187
Icos	0.521	1.009	0.466	0.992	0.728	0.618
Id2	0.918	1.076	0.984	0.987	0.967	0.913
Ifng	1.300	1.104	1.516	1.323	1.492	1.351
Igsf6	1.564	0.917	1.100	0.755	1.381	1.210
Ikzf2	0.623	0.770	0.533	0.668	0.896	0.581

Il12b	0.695	0.945	0.991	0.816	2.235	0.844
Il12rb2	0.762	0.681	0.588	0.663	1.153	0.812
Il13	1.251	2.119	1.885	2.513	3.717	2.414
Il13ra1	1.607	0.777	1.484	0.944	1.152	1.069
Il17a	0.703	0.353	0.600	0.683	0.759	0.986
Il17re	1.641	1.221	2.015	1.573	1.544	1.148
Il18	1.692	1.710	1.353	1.123	1.521	1.336
Il18r1	1.204	1.230	1.122	0.934	1.053	1.029
Il18rap	1.082	1.317	0.589	0.672	0.968	1.508
Il1r1	0.575	0.837	0.827	0.764	0.703	0.848
Il1r2	1.458	3.719	1.821	0.974	1.628	3.206
Il1rl1	0.506	0.718	0.709	0.976	0.844	0.761
Il2	2.488	0.723	1.564	0.667	2.298	1.428
Il21	1.910	1.165	1.100	0.682	2.784	1.242
Il2ra	1.035	0.934	1.086	0.871	1.341	0.733
Il4	2.083	1.665	1.804	1.380	2.069	0.860
Il4ra	1.734	2.054	1.365	1.676	1.345	1.735
Il5	0.567	1.701	0.371	0.840	2.141	2.642
Il9	0.215	3.524	0.111	4.618	0.429	5.840
Irf1	1.046	1.099	1.068	0.985	0.967	0.885
Irf4	0.739	1.165	0.747	0.776	1.141	0.654
Irf8	1.283	1.395	1.070	1.165	1.005	0.927
Jak1	1.514	1.038	1.476	1.043	1.323	1.063
Lrrc32	0.904	2.533	0.758	1.657	0.772	1.642
Maf	1.097	1.386	1.022	1.170	0.922	1.237
Myb	1.215	0.888	1.289	0.857	1.577	0.727
Nfatc1	1.381	1.007	1.261	0.933	1.412	0.970
Nfatc2	0.938	0.961	0.995	0.918	0.827	0.885
Nfatc2ip	1.528	1.342	1.439	1.098	1.268	1.047
Nr4a1	1.624	1.431	1.592	1.061	1.384	1.027
Nr4a3	0.235	0.930	0.206	0.734	0.208	1.081
Perp	0.981	1.163	1.693	0.591	0.845	1.408
Pkd2	0.828	1.424	0.775	1.284	0.901	1.644
Pou2f2	1.429	1.462	1.456	1.105	1.397	0.976
Pparg	0.635	1.240	0.904	1.053	0.610	1.032
Rel	1.834	1.102	1.565	0.834	1.451	0.834
Rora	1.603	1.238	1.792	1.012	1.200	1.043
Rorc	1.159	1.515	1.361	1.278	1.566	1.829
Runx1	1.373	1.035	1.266	1.027	1.345	1.097
Runx3	0.687	1.466	0.660	1.180	0.696	1.171
Socs1	5.505	1.069	1.424	1.211	1.531	1.079
Socs5	2.017	0.794	1.521	1.093	1.203	1.245
Stat1	1.656	0.722	2.029	0.833	1.841	0.768
Stat4	0.929	1.478	1.052	1.066	1.154	0.849
Stat6	1.647	1.422	1.119	1.200	1.117	1.142
Tbx21	0.404	1.571	0.296	0.811	0.770	1.193

Tgif1	1.452	0.743	0.940	0.664	1.092	1.285
Tlr4	1.469	0.736	1.030	0.768	0.944	1.151
Tlr6	0.550	1.306	0.675	0.873	0.650	1.290
Tmed1	0.881	1.407	0.976	1.154	0.796	1.321
Tnf	0.506	0.997	0.503	1.374	0.399	0.985
Tnfrsf9	0.963	1.283	1.083	1.067	1.505	1.006
Tnfsf11	1.552	0.820	1.164	0.877	1.560	0.480
Trp53inp1	0.674	0.921	0.545	0.679	0.712	0.863
Uts2	3.795	3.326	0.644	2.161	5.583	3.080
Zbtb7b	1.560	0.916	1.459	0.910	1.585	1.120
Zeb1	2.058	2.361	1.918	2.349	1.862	3.430

Lymph node samples were collected from mice treated with calcitriol or its analogs and control group receiving vehicle on the days 21 and 28 (after inoculation with 4T1 cells). Real-time PCR screening was performed using *Mouse T Helper Cell Differentiation RT² Profiler Array* (Qiagen. Hilden. Germany) including 84 key genes and 5 housekeeping genes in the set (Table S1). Data presented as a mean relative quantification (RQ) values. Fold-change (RQ) of target cDNA was defined using double delta Ct method in reference to beta-2 microglobulin (*B2m*), glyceraldehyde-3-phosphate dehydrogenase (*Gapdh*) and heat shock protein 90 alpha, class B member 1 (*Hsp90ab1*). Next the results were adjusted to the values obtained for the control group within the day 14 or 28th of the experiment for each treatment group. Data analysis was acquired using Qiagen online software suitable for purchased kit (Qiagen. Hilden. Germany). PCR amplification cycles were as follows 95 °C for 10 s and 58 °C for 45 s (50 cycles). We used 0.5 µg of cDNA (6 mice pooled per group) for a single reaction.

Table S6. The expression of genes associated with tumor invasion or metastasis evaluated in 4T1 lung tissue after treatment with calcitriol or its analogs.

GENE	Control		Calcitriol		PRI-2191		PRI-2205	
	D14	D28	D14	D28	D14	D28	D14	D28
Adamts1	0.1021	6.9524	4.0763	0.2026	2.6365	0.3105	0.2591	0.1217
Aldh3a1	0.1081	3.4041	5.2648	0.1759	1.8660	0.4573	0.0288	0.1350
Angpt1	0.0175	5.3426	14.3275	0.4190	0.8559	0.0481	1.7566	0.0040
Angpt14	0.0717	2.8635	1.6292	0.1172	0.1740	0.0533	0.1094	0.0794
Casp8	0.1609	4.4852	17.1495	0.1560	0.8823	0.5039	0.2987	0.4391
Cdh2	0.1950	4.4482	3.0244	0.0991	0.7191	1.3455	1.5076	0.0743
Cdh6	0.6451	4.0217	11.8376	0.3184	2.3374	0.3950	1.1163	0.9422
Col4a2	0.2231	14.0953	9.9344	2.2378	2.1428	0.2715	0.8251	1.1612
Col6a1	0.5293	1.9947	1.4182	0.0779	0.4406	0.2262	0.4336	0.4766
Cst7	0.6212	5.4917	5.7955	15.5825	5.5213	3.8085	1.8882	13.6300
Ctgf	0.0301	0.5168	0.4273	0.0283	0.0541	0.0781	0.1498	0.0664
Ctsb	0.2542	3.1115	8.4300	0.2027	0.3488	0.1780	0.6045	1.0043
Ctsl1	0.0322	2.0009	3.4083	0.0294	0.0803	0.2343	0.9123	0.0512
Cxcl1	0.9050	9.8453	9.4692	6.1423	6.4151	3.6930	2.2256	21.3126
Drg1	0.0147	0.8098	0.1756	0.0353	0.0795	0.0511	0.0683	0.0875
Flt1	0.0923	1.6656	9.5041	0.2455	0.7573	0.3315	1.1368	0.1928
Gpi	0.6091	7.4262	9.5362	24.3252	4.8662	4.1971	1.8743	10.1971
Gsn	0.9534	6.6025	9.7069	1.0058	4.4323	0.5571	1.6766	0.8818
Id1	0.3805	4.7843	13.5532	0.3334	1.2883	0.4716	0.5435	0.3495

Isg20	0.1123	1.8314	7.3532	0.0350	0.2886	0.1615	0.2314	0.1935
Map2k4	0.1032	3.2856	1.4784	0.1501	0.5141	0.3991	0.0467	0.2073
Mcam	0.0836	0.2120	0.4795	0.0286	0.1504	0.0245	0.3727	0.0273
Metap2	14.3950	9.1724	9.7487	4.6765	9.9921	0.0137	35.8166	36.8244
Mmp11	0.7830	2.7765	22.9619	0.3264	2.9887	0.3795	1.1443	0.7011
Mmp13	0.0420	1.7269	0.7483	0.1373	0.2746	3.5925	0.0657	0.3389
Mmp14	1.0173	1.6715	7.7031	0.1365	1.4630	0.0857	0.4762	1.0691
Mmp2	0.1803	0.7334	12.0421	0.1514	0.4155	0.0196	0.5275	0.1447
Nedd9	2.7636	8.6074	1.0877	0.0755	0.2595	0.0271	0.3969	0.1664
Nf2	0.1619	9.4709	1.4281	11.2397	0.2890	0.1596	0.2174	0.1802
Nme2	0.6354	9.8238	20.6674	1.0054	1.9418	0.2143	0.0024	0.0029
Pax5	0.8642	21.6493	9.1668	1.5501	6.4063	0.2890	1.7699	1.6823
Pdgfa	0.2329	21.1707	5.2079	0.2951	1.1809	0.0557	0.3843	0.6058
Plaur	0.1704	9.6868	0.8387	0.4348	0.1407	0.0939	0.2691	0.5652
Serpin1	0.76645	35.42515	6.8383	2.2784	1.6462	0.14405	1.1322	3.2355
Sparc	0.04625	0.1872	3.94765	0.02625	0.1818	0.0016	0.0625	0.0975
Spp1	0.14845	0.4796	0.4975	0.162	0.721	2.6722	2.266	2.2923
Src	0.1781	1.7142	2.14515	0.1282	0.3674	0.0278	0.2587	0.3649
Tgfb1	0.287	1.49485	0.952	0.2028	1.03565	0.44	0.1180	0.4342
Timp1	0.1075	32.78185	0.32545	2.77745	0.2614	0.41765	0.2206	1.0214
Timp2	0.1829	0.13365	10.02855	2.58465	0.0857	0.10945	0.1581	0.0648
Timp3	0.3003	0.1151	26.33775	0.0773	0.55415	0.0976	0.0228	0.0862
Timp4	0.3497	0.21445	0.62505	0.12745	1.1912	0.09305	0.1347	0.0988
Vegfa	0.64185	2.3577	10.97125	2.1168	0.9339	0.2302	0.526	0.229

Lung specimens were collected on the days 14 and 28 (after inoculation with 4T1 cells) from mice treated with calcitriol or its analogs and control group receiving vehicle. Real-time PCR screening was performed using the mouse tumor invasion/metastasis PCR array library (MTIM-1). From 88 genes available in this array (Table S2) the expression for 45 genes was not detected in 4T1 lung tissue. Data presented as a mean relative quantification (RQ) values (calculated from duplicate). Fold-change (RQ) of target cDNA was determined by calculating the differences in ΔΔCT values in reference to ribosomal protein L13A (*Rpl13a*) and adjusted to the values obtained for the untreated mice (named D0) for each treatment group. Data analysis was performed by DataAssist v 3.01 software. All PCR amplification cycles were performed at 95 °C for 10 s and 58 °C for 45 s (50 cycles). We used 25 ng of cDNA for a single reaction and each test was performed in duplicate.

Table S7. The number of lung metastases in mice bearing 4T1 mammary gland cancer cells and treated with calcitriol or its analogs: PRI-2191 and PRI-2205.

Day of experiment	No of metastases [median (min-max)]			
	**Control**	**Calcitriol**	**PRI-2191**	**PRI-2205**
**D21**	3 (1.0-4.5)	3 (0-6.0)	3 (0.5-6.0)	3.5 (0.5-12.0)*
**D28**	11 (3.0-68.0)	14 (6.0-29.0)	25 (13.0-60.0)*	17.25 (0-83.0)

D33	12 (5.0-25.5)	18 (3.0-34.0)	18.5 (4.5-63.0)	19 (4.0-44.0)
**Day of experiment**	**Score for metastases [median (min-max)]**			
	**Control**	**Calcitriol**	**PRI-2191**	**PRI-2205**
**D14**	0 (0-1)	0 (0-0)	0.5 (0-1)	0 (0-1)
**D21**	1 (0-2)	1 (1-1)	1 (0-2)	1 (0-1)
**D28**	2 (1-4)	2 (1-3)	3 (1-4)	4 (1-4)
**D33**	1 (0-4)	4 (4-4)*	4 (3-4)*	4 (3-4)*

*p<0.05. Statistical analysis: Kruskal-Wallis multiple comparison test. Number of lung metastases was counted in lungs macroscopically by two independent examiners. Score for metastases was performed by pathologist in H&E stained lungs on days 14, 21, 28 and 33: 0, no metastasis detected; +, 1-3 metastatic foci; ++, 4-7 foci; +++, 8-10 foci; ++++, >10 metastatic foci in lungs. Mice were inoculated orthotopically with 4T1 cells on day 0. From day 7 (7 days after tumor inoculation), vitamin D analogs were administered subcutaneously (s.c.) thrice a week. The single dose of compounds were as follows: calcitriol, 0.5 µg/kg; PRI-2191, 1.0 µg/kg; and PRI-2205, 10.0 µg/kg. Number of mice: 9-13 per group. [Data from: Anisiewicz, A. *et al.* Unfavorable effect of calcitriol and its low-calcemic analogs on metastasis of 4T1 mouse mammary gland cancer. *Int. J. Oncol.* **52,** 103–126 (2018)].

Table S8. List of genes included in *Mouse T Helper Cell Differentiation RT² Profiler Array.*

Symbol	Description
Asb2	Ankyrin repeat and SOCS box-containing 2
Cacna1f	Calcium channel, voltage-dependent, alpha 1F subunit
Ccl11	Chemokine (C-C motif) ligand 11
Ccl5	Chemokine (C-C motif) ligand 5
Ccl7	Chemokine (C-C motif) ligand 7
Ccr3	Chemokine (C-C motif) receptor 3
Ccr4	Chemokine (C-C motif) receptor 4
Ccr6	Chemokine (C-C motif) receptor 6
Cebpb	CCAAT/enhancer binding protein (C/EBP), beta
Chd7	Chromodomain helicase DNA binding protein 7
Csf2	Colony stimulating factor 2 (granulocyte-macrophage)
Fasl	Fas ligand (TNF superfamily, member 6)
Fosl1	Fos-like antigen 1
Foxp3	Forkhead box P3
Gata3	GATA binding protein 3
Gata4	GATA binding protein 4
Gfi1	Growth factor independent 1
Ptgdr2	G protein-coupled receptor 44
Havcr2	Hepatitis A virus cellular receptor 2
Hopx	HOP homeobox
Hoxa10	Homeobox A10
Hoxa3	Homeobox A3

Icos	Inducible T-cell co-stimulator
Id2	Inhibitor of DNA binding 2
Ifng	Interferon gamma
Igsf6	Immunoglobulin superfamily, member 6
Ikzf2	IKAROS family zinc finger 2
Il12b	Interleukin 12B
Il12rb2	Interleukin 12 receptor, beta 2
Il13	Interleukin 13
Il13ra1	Interleukin 13 receptor, alpha 1
Il17a	Interleukin 17A
Il17re	Interleukin 17 receptor E
Il18	Interleukin 18
Il18r1	Interleukin 18 receptor 1
Il18rap	Interleukin 18 receptor accessory protein
Il1r1	Interleukin 1 receptor, type I
Il1r2	Interleukin 1 receptor, type II
Il1rl1	Interleukin 1 receptor-like 1
Il2	Interleukin 2
Il21	Interleukin 21
Il2ra	Interleukin 2 receptor, alpha chain
Il4	Interleukin 4
Il4ra	Interleukin 4 receptor, alpha
Il5	Interleukin 5
Il9	Interleukin 9
Irf1	Interferon regulatory factor 1
Irf4	Interferon regulatory factor 4
Irf8	Interferon regulatory factor 8
Jak1	Janus kinase 1
Lrrc32	Leucine rich repeat containing 32
Maf	Avian musculoaponeurotic fibrosarcoma (v-maf) AS42 oncogene homolog
Myb	Myeloblastosis oncogene
Nfatc1	Nuclear factor of activated T-cells, cytoplasmic, calcineurin-dependent 1
Nfatc2	Nuclear factor of activated T-cells, cytoplasmic, calcineurin-dependent 2
Nfatc2ip	Nuclear factor of activated T-cells, cytoplasmic, calcineurin-dependent 2 interacting protein
Nr4a1	Nuclear receptor subfamily 4, group A, member 1
Nr4a3	Nuclear receptor subfamily 4, group A, member 3
Perp	PERP, TP53 apoptosis effector
Pkd2	Polycystic kidney disease 2
Pou2f2	POU domain, class 2, transcription factor 2
Pparg	Peroxisome proliferator activated receptor gamma
Rel	Reticuloendotheliosis oncogene
Rora	RAR-related orphan receptor alpha
Rorc	RAR-related orphan receptor gamma
Runx1	Runt related transcription factor 1
Runx3	Runt related transcription factor 3
Socs1	Suppressor of cytokine signaling 1

Socs5	Suppressor of cytokine signaling 5
Stat1	Signal transducer and activator of transcription 1
Stat4	Signal transducer and activator of transcription 4
Stat6	Signal transducer and activator of transcription 6
Tbx21	T-box 21
Tgif1	TGFB-induced factor homeobox 1
Tlr4	Toll-like receptor 4
Tlr6	Toll-like receptor 6
Tmed1	Transmembrane emp24 domain containing 1
Tnf	Tumor necrosis factor
Tnfrsf9	Tumor necrosis factor receptor superfamily, member 9
Tnfsf11	Tumor necrosis factor (ligand) superfamily, member 11
Trp53inp1	Transformation related protein 53 inducible nuclear protein 1
Uts2	Urotensin 2
Zbtb7b	Zinc finger and BTB domain containing 7B
Zeb1	Zinc finger E-box binding homeobox 1

Table S9. List of genes evaluated using mouse tumor invasion/metastasis PCR array library (MTIM-1).

Symbol	Description
Adamts1	ADAM metallopeptidase
Aldh3a1	Aldehyde dehydrogenase 3 family, memberA1
Angpt1	Angiopoietin 1
Angptl4	Angiopoietin-like 4
Casp8	Caspase 8, apoptosis-related cysteine peptidase
Ccne2	Cyclin E2
Ccr7	Chemokine (C-C motif) receptor 7
Cd44	CD44 molecule (Indian blood group)
Cd82	CD82 antigen
Cdh1	Cadherin 1, type 1, E-cadherin (epithelial)
Cdh11	Cadherin 11, type 2, OB-cadherin (osteoblast)
Cdh2	Cadherin 2, type 1, N-cadherin
Cdh6	Cadherin 6
Cldn7	Claudin 7
Col1a1	Collagen, type I, alpha 1
Col4a2	Collagen, type IV, alpha 2
Col6a1	Collagen, type VI, alpha 1
Csf1	Colony stimulating factor 1
Csf2	Colony stimulating factor 2
Csf3	Colony stimulating factor 3
Cst7	Cystatin F (leukocystatin)
Ctgf	Connective tissue growth factor
Ctsb	Cathepsin B
Ctsd	Cathepsin D
Ctsk	Cathepsin K
Ctsl1	Cathepsin L-like 3

Cxcl1	Chemokine (C-X-C motif) ligand 1
Cxcl13	Chemokine (C-X-C motif) ligand 13
Cxcr4	Chemokine (C-X-C motif) receptor 4
Cxcr6	Chemokine (C-X-C motif) receptor 6
Drg1	Developmentally regulated GTP binding protein 1
Ereg	Epiregulin
Fgf8	Fibroblast growth factor 8 (androgen-induced)
Flt1	Fms-related tyrosine kinase
Flt4	Fms-related tyrosine kinase 4
Gpi	Glucose phosphate isomerase
Gsn	Gelsolin (amyloidosis, Finnish type)
Hgf	Hepatocyte growth factor (hepapoietin A; scatter factor)
Hif1a	Hypoxia inducible factor 1, alpha subunit
Hmgb1	High-mobility group box 1
Id1	Inhibitor of DNA binding 1
Il13ra2	Interleukin 13 receptor, alpha 2
Isg20	Interferon stimulated exonuclease gene 20kDa
Jag1	Jagged 1 (Alagille syndrome)
Kiss1	KiSS-1 metastasis-suppressor
Klrc2	Killer cell lectin-like receptor subfamily C, member 2
Kynu	Kynureninase (L-kynurenine hydrolase)
Ltbp1	Latent transforming growth factor beta binding protein 1
Map2k4	Mitogen-activated protein kinase kinase 4
Map2k5	Mitogen-activated protein kinase kinase 5
Map2k7	Mitogen-activated protein kinase kinase 7
Mcam	Melanoma cell adhesion molecule
Met	Met proto-oncogene (hepatocyte growth factor receptor)
Metap2	Methionyl aminopeptidase 2
Mmp1	Matrix metallopeptidase 1 (interstitial collagenase)
Mmp10	Matrix metallopeptidase 10 (stromelysin 2)
Mmp11	Matrix metallopeptidase 11 (stromelysin 3)
Mmp13	Matrix metallopeptidase 13 (collagenase 3)
Mmp14	Matrix metallopeptidase 14 (membrane-inserted)
Mmp2	Matrix metallopeptidase 2
Mmp7	Matrix metallopeptidase 7 (matrilysin, uterine)
Myc	V-myc myelocytomatosis viral oncogene homolog
Nedd9	Neural precursor cell expressed, dev. down-regulated 9
Nf2	Neurofibromin 2 (merlin)
Nme1	Non-metastatic cells 1, protein (NM23A)
Nme2	Non-metastatic cells 2, protein
Nme4	Non-metastatic cells 4, protein
Pax5	Paired box 5
Pdgfa	Platelet-derived growth factor alpha polypeptide
Plaur	Plasminogen activator, urokinase receptor
Ptgs2	Prostaglandin-endoperoxide synthase 2
Runx1	Runt-related transcription factor 1
Serpine1	Serpin peptidase inhibitor, clade E
Serpinb5	Serpin peptidase inhibitor, clade B5

Sox4	SRY (sex determining region Y)-box 4
Sparc	Secreted protein, acidic, cysteine-rich (osteonectin)
Spp1	Secreted phosphoprotein 1
Src	V-src sarcoma viral oncogene homolog (avian)
Tff1	Trefoil factor 1
Tgfb1	Transforming growth factor, beta 1
Timp1	TIMP metallopeptidase inhibitor 1
Timp2	TIMP metallopeptidase inhibitor 2
Timp3	TIMP metallopeptidase inhibitor 3
Timp4	TIMP metallopeptidase inhibitor 4
Tnc	Tenascin C (hexabrachion)
Tp53	Tumor protein p53
Vegfa	Vascular endothelial growth factor A
Vegfb	Vascular endothelial growth factor B
Actb	Actin, beta
B2m	Beta-2-microglobulin
Gapd	Glyceraldehyde-3-phosphate dehydrogenase
Gusb	Glucuronidase, beta
Hprt1	Hypoxanthine phosphoribosyltransferase 1
Pgk	Phosphoglycerate kinase 1
Ppia	Peptidylprolyl isomerase A
Rpl13a	Ribosomal protein L13a
